# MOBP and HIP1 in multiple system atrophy: New α‐synuclein partners in glial cytoplasmic inclusions implicated in the disease pathogenesis

**DOI:** 10.1111/nan.12688

**Published:** 2021-01-19

**Authors:** Conceição Bettencourt, Yasuo Miki, Ignazio S. Piras, Rohan de Silva, Sandrine C. Foti, Joshua S. Talboom, Tamas Revesz, Tammaryn Lashley, Robert Balazs, Emmanuelle Viré, Thomas T. Warner, Matt J. Huentelman, Janice L. Holton

**Affiliations:** ^1^ Queen Square Brain Bank for Neurological Disorders UCL Queen Square Institute of Neurology London UK; ^2^ Department of Clinical and Movement Neurosciences UCL Queen Square Institute of Neurology London UK; ^3^ Department of Neuropathology Institute of Brain Science Hirosaki University Graduate School of Medicine Hirosaki Japan; ^4^ Neurogenomics Division Translational Genomics Research Institute Phoenix AZ USA; ^5^ Reta Lila Weston Institute UCL Queen Square Institute of Neurology London UK; ^6^ Department of Neurodegenerative Disease UCL Queen Square Institute of Neurology London UK; ^7^ Institute of Prion Diseases MRC Prion Unit at UCL London UK

**Keywords:** DNA methylation‐gene expression correlations, α‐synuclein protein interactors, glial cytoplasmic inclusions, MSA pathogenesis, myelination, clathrin‐dependent endocytosis

## Abstract

**Aims:**

Multiple system atrophy (MSA) is a fatal neurodegenerative disease. Similar to Parkinson's disease (PD), MSA is an α‐synucleinopathy, and its pathological hallmark consists of glial cytoplasmic inclusions (GCIs) containing α‐synuclein (SNCA) in oligodendrocytes. We previously identified consistent changes in myelin‐associated oligodendrocyte basic protein (*MOBP*) and huntingtin interacting protein 1 (*HIP1*) DNA methylation status in MSA. We hypothesized that if differential DNA methylation at these loci is mechanistically relevant for MSA, it should have downstream consequences on gene regulation.

**Methods:**

We investigated the relationship between *MOBP* and *HIP1* DNA methylation and mRNA levels in cerebellar white matter from MSA and healthy controls. Additionally, we analysed protein expression using western blotting, immunohistochemistry and proximity ligation assays.

**Results:**

We found decreased *MOBP* mRNA levels significantly correlated with increased DNA methylation in MSA. For *HIP1*, we found a distinct relationship between DNA methylation and gene expression levels in MSA compared to healthy controls, suggesting this locus may be subjected to epigenetic remodelling in MSA. Although soluble protein levels for MOBP and HIP1 in cerebellar white matter were not significantly different between MSA cases and controls, we found striking differences between MSA and other neurodegenerative diseases, including PD and Huntington's disease. We also found that MOBP and HIP1 are mislocalized into the GCIs in MSA, where they appear to interact with SNCA.

**Conclusions:**

This study supports a role for DNA methylation in downregulation of *MOBP* mRNA in MSA. Most importantly, the identification of MOBP and HIP1 as new constituents of GCIs emphasizes the relevance of these two loci to the pathogenesis of MSA.

## Graphical abstract

By investigating loci that have shown significant DNA methylation alterations in multiple system atrophy (MSA), this study identified myelin‐associated oligodendrocyte basic protein (MOBP) and huntingtin interacting protein 1 (HIP1) as new constituents of glial cytoplasmic inclusions (highlighted by arrowheads in b and d) and α‐synuclein interactors, thus strengthening the potential role of these two loci in MSA pathogenesis.

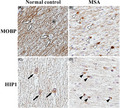

## INTRODUCTION

Multiple system atrophy (MSA) is a fatal neurodegenerative disease and its aetiology remains elusive. As with Parkinson's disease (PD), MSA is an α‐synucleinopathy, and its pathological hallmark consists of glial cytoplasmic inclusions (GCIs) containing fibrillar α‐synuclein (SNCA) in oligodendrocytes.^1^, ^2^, ^3^ As the number of GCIs correlates with the disease duration and severity of neurodegeneration, the burden of GCIs is thought to be an important factor in the pathogenesis of MSA.^4^ In MSA, the regional distribution of pathological changes underlies the clinical symptoms. MSA patients with a prominent parkinsonian movement disorder have predominantly striatonigral degeneration (SND subtype), while those with prominent cerebellar signs have more severe olivopontocerebellar atrophy (OPCA subtype).^4^, ^5^ However, at post‐mortem examination, cases often show an equal distribution of neurodegeneration in SND and OPCA regions (mixed subtype, SND = OPCA).^4^

DNA methylation is an epigenetic modification that consists of the covalent addition of methyl groups to nucleotide bases, often at CpG motifs. Although not all gene regulatory elements are functionally methylation dependent, DNA methylation is a regulatory mechanism that plays a major role in development,^6^, ^7^ disease susceptibility^8^, ^9^ and the response to environmental conditions.^10^, ^11^ In support of the role of DNA methylation in neurodegenerative diseases, there has been an increasing number of epigenome‐wide association studies (EWAS), including our study on MSA,^12^ showing links between DNA methylation levels and disease (e.g. ^13^, ^14^). Our MSA EWAS^12^ identified *MOBP* (myelin associated oligodendrocyte basic protein) and *HIP1* (Huntingtin Interacting Protein 1) among the most differentially methylated loci in MSA when compared to healthy controls. These two genes are particularly interesting candidates for MSA pathogenesis as they are more highly expressed in oligodendrocytes when compared to the other major cell types in the brain. We hypothesized that if differential DNA methylation at these loci is mechanistically relevant for the disease, it should also have downstream consequences on gene regulation. To test this hypothesis, we have herein investigated whether in MSA there are downstream changes in *MOBP* and *HIP1* at the mRNA and protein levels, and also in protein localization, in the cerebellar white matter, a brain region that is severely affected by GCI burden in MSA OPCA and mixed pathological subtypes. In the present study, we have shown decreased *MOBP* mRNA expression levels in MSA significantly correlated with increased DNA methylation levels at the gene promoter. For *HIP1*, we found a complex relationship between DNA methylation and gene expression levels suggestive of epigenetic remodelling in MSA. Regarding the soluble protein levels, although we observe comparable levels between MSA and controls, we found striking differences between MSA and other neurodegenerative diseases. Most interestingly, we found MOBP and HIP1 to be mislocalized into the GCIs in MSA, where they appear to interact with SNCA, emphasizing their relevance to MSA pathogenesis.

## MATERIALS AND METHODS

### Demographic characteristics of post‐mortem brain donors

All tissue came from brains donated to the Queen Square Brain Bank for Neurological Disorders. Frozen white matter tissue from the cerebellum was used for DNA methylation profiling, RNA sequencing (RNAseq) and western blotting (Table S1.1). Frozen occipital lobe white matter tissue from MSA and controls was also analysed by western blotting. Additionally, formalin‐fixed paraffin‐embedded (FFPE) sections from the cerebellar hemisphere, mid brain or frontal cortex, were used for immunohistochemistry and proximity ligation assays (PLAs). For western blotting analysis, cerebellar hemispheric white matter samples from PD, progressive supranuclear palsy (PSP) and Huntington's disease (HD) cases were used as comparison to the MSA and controls. There were no significant differences in age at death or post‐mortem interval across groups (Table S1.2). PD was chosen as another α‐synucleinopathy, PSP as a disease that presents oligodendrocyte inclusions and has variants in *MOBP* associated with disease risk,^15^, ^16^ HD as a disease in which the mutant protein huntingtin (HTT) is a known interactor of HIP1.^17^

### DNA methylation and gene expression analyses

Our recent study investigating DNA methylation profiling in MSA revealed *MOBP* and *HIP1* among the most differentially methylated loci in cerebellar white matter.^12^ In another recent study,^18^ we also investigated changes in gene expression in cerebellar white matter as well as in microdissected oligodendrocytes of MSA cases and healthy controls, by performing RNA sequencing (RNAseq). To gain insights into whether DNA methylation has a regulatory role on *MOBP* and *HIP1* gene expression levels, we analysed data from 14 MSA cases and 10 healthy controls for which we had both DNA methylation^12^ and gene expression data.^18^

The genome‐wide DNA methylation profiles used in this study were obtained as previously described.^12^ Briefly, DNA methylation data were generated using the Infinium HumanMethylationEPIC BeadChip (Illumina), and analysed using R Bioconductor packages as previously described.^12^ Beta values were used to estimate the methylation levels of each CpG site using the ratio of intensities between methylated and unmethylated alleles, and *M*‐values (logistic transformation of the beta values) were used for all statistical analysis. The RNAseq data were generated and processed as described by Piras et al.^18^ For the correlation analysis between DNA methylation and gene expression levels, we used M‐values adjusted for possible confounding factors, including age, gender, post‐mortem interval, neuronal proportions, batch effect and surrogate variables (as previously described^12^). The raw RNA counts were transformed using the variance stabilizing transformation using the R‐package DESeq2 (25516281), and then adjusted for confounding factors (age, sex and post‐mortem interval) using the function removeBatchEffect as implemented in the R package limma (25605792). All CpG methylation sites mapping to *MOBP* (*N* = 29 CpGs) and *HIP1* (*N* = 68 CpGs) that passed quality control were included in this analysis (Table S2). *M*‐values from each CpG site were compared with the RNA levels of the corresponding gene by determining Pearson's correlation coefficients using the *cor.test* function as implemented in R. We considered as level of significance *p* < 0.05. Additionally, we conducted a permutation analysis to test whether the opposite correlation signals between MSA cases and controls observed for *HIP1* were due to chance. Specifically, 1000 genes were randomly selected, we then computed the methylation‐expression correlation coefficients for MSA and controls and counted for each gene how many times the number of opposite correlation signals was equal or greater than that detected for *HIP1*, using as cut‐off *r* ≥ |0.4|. We considered a 5% threshold to attribute the opposite correlation signals as an effect due to chance (≥5%).

To investigate the expression levels of *HIP1* and *MOBP* in major brain cell types from healthy controls, we have used data from Zhang et al.,^19^ including samples from hippocampus, temporal lobe and foetal cortex. Raw data were downloaded from Sequencing Reads Archive (#SRP064454), and the pseudoalignment was conducted with Kallisto v0.46.1. Finally, RNA counts were normalized with DESeq2 v1.26.0 to generate plots of RNA expression levels across the different cell types.

### Protein homogenization and western blotting

Flash frozen cerebellar hemispheric white matter, which is severely affected in MSA mixed subtype, was carefully dissected from control (*N* = 6), MSA mixed (*N* = 6), PD (*N* = 6), PSP (*N* = 6) and HD (*N* = 5) cases. Additionally, occipital lobe white matter, which is minimally affected in MSA mixed subtype, was carefully dissected from all control (*N* = 5) and MSA mixed cases (*N* = 6) that had flash frozen tissue available. The tissue was homogenized in a high‐salt lysis buffer (50 mM Tris‐HCl pH 7.4, 175 mM NaCl, 1% Triton‐X100 with Complete® protease and PhosStop® phosphatase inhibitor tablets (Roche) using the Precellys 24 with tissue homogenizing CKMix ceramic beads (Bertin Technologies). The resulting homogenate was then spun at 4000 rpm for 10 min at 4°C, and the supernatant was recovered and stored at −20°C. Protein concentration was determined by a Bicinchoninic acid assay (BCA) following the manufacturer's recommendations (BCA; Thermo Scientific Massachusetts). For the sodium dodecyl sulphate–polyacrylamide gel electrophoresis, 20–25 µg of each sample was loaded into a 4%–12% Criterion XT Bis‐Tris Gel (Bio‐Rad) and run in XT MES (for MOBP) or XT MOPS (for HIP1) running buffer (Bio‐Rad) at 200 V. Each sample was run in duplicate over different gels, each containing control, MSA, PD, PSP and HD samples on one gel for the cerebellar white matter samples, or each containing control and MSA for the occipital lobe samples. The gels were then transferred to Trans‐Blot Turbo Mini Nitrocellulose Transfer Packs using a Trans‐Blot Turbo system (Bio‐Rad). The membrane was then blocked in 5% semi‐dry powdered milk/phosphate‐buffered saline (PBS) for 1 h at RT on a shaker. The membranes were incubated with a primary antibody (anti‐MOBP 1:250, Atlas Antibodies HPA035152; anti‐HIP1 1:2000; Abcam ab181238) diluted in 2% BSA/PBS‐0.1%Tween (PBS‐T) overnight at 4 °C. The membranes were washed in PBS‐T and incubated with respective LiCOR IRDye 680RD/800CW secondary antibodies for an hour on the shaker. The membranes were then washed three times with PBS‐T, followed by a fourth wash in PBS. Blots were scanned and fluorescent images acquired using a LiCor Odyssey Fc scanner with the Image Studio software (LI‐COR). Each protein was normalized to β‐actin (anti‐β‐actin 1:5000; Sigma A1978) and then to one internal control sample in each gel before averaging the duplicates. For MOBP, we quantified the dominant band of 9.5 kDa and additionally the area corresponding to bands between 21 and 23.4 kDa. Total MOBP was estimated as 9.5 kDa + 21–23.4 kDa bands, and MOBP isoform ratio as 21–23.4 kDa/9.5 kDa bands. For HIP1, we quantified the band of around 115 kDa. These values are represented as boxplots in Figures 3 and 4 for the cerebellar white matter samples (full blots are shown in Figures S1 and S2), and Figures S3 and S4 for the occipital lobe white matter samples.

Differences across groups (controls, MSA, PD, PSP and HD for cerebellar samples, and controls and MSA for occipital samples) were performed using Kruskal‐Wallis rank sum test, followed by pairwise comparisons between groups using the Wilcoxon rank‐sum test with Benjamini–Hochberg (BH) adjustment for multiple testing. This statistical analysis was performed with the R suit and adjusted *p* < 0.05 were considered as significant.

Spearman's correlation coefficients, obtained using the *rcorr* function as implemented in R, were used to evaluate the co‐expression patterns of MOBP isoforms and HIP1 in MSA and controls. Spearman's correlation coefficients were also used to investigate the relationship between MOBP isoforms and HIP1 protein expression levels and MSA disease traits, including disease onset, disease duration, cerebellar GCI burden and Purkinje cell loss (assessed as previously described^12^). We considered as level of significance *p* < 0.05.

### Immunohistochemical staining

Ten cases were utilized to investigate tissue expression patterns of MOBP and HIP1. These included MSA cases (*N* = 5) and healthy controls (*N* = 5). Eight‐micrometre‐thick sections were cut from the cerebellar hemispheric FFPE blocks of patients with MSA and healthy controls and immunostained using a standard avidin‐biotin‐peroxidase complex method with di‐aminobenzidine as the chromogen. The antibodies used in this study were MOBP (Atlas Antibodies HPA035152, 1:200; Bioss Antibodies BS‐11184R, 1:100) and HIP1 (Abcam ab181238, 1:100; Novus Biologicals NB300‐203, 1:2000). Heat antigen retrieval pre‐treatment was used prior to application of the primary antibodies unless otherwise specified. The samples were mounted and examined using a light microscope. Additionally, three cases per disease control group (PD *N* = 3, PSP *N* = 3, and HD *N* = 3) were used to investigate the disease specificity of expression patterns of MOBP and HIP1. Mid brain was investigated in PD, and the frontal cortex in PSP and HD. FFPE tissue sections were processed and stained as described above for the cerebellar samples. FFPE tissue sections were also stained with mouse anti‐SNCA (Abcam ab1903, 1:1000), mouse anti‐AT8 (Invitrogen MN1020, 1:600) and mouse anti‐IC2 (Chemicon MAB1574, 1:1000, formic acid pre‐treatment) for PD midbrain, and frontal cortex for PSP and HD respectively.

### Proximity ligation assay

A PLA was performed according to the manufacturer's instructions using Duolink kit supplied by Sigma (Sigma‐Aldrich Company Ltd). Four tissue sections from MSA cases were examined for PLA. Two were utilized as negative controls without primary antibodies. Mouse anti‐SNCA (Abcam ab1903, 1:1000) and rabbit anti‐MOBP (Atlas Antibodies HPA035152, 1:200) or anti‐HIP1 (Abcam ab181238, 1:100) antibodies were used as primary antibodies for PLA. As reported previously,^20^ anti‐SNCA and MOBP or HIP1 antibodies were applied to the samples and incubated for 1h at room temperature. PLA probes (Minus and Plus) detecting each antibody were added to the samples for 1 h at 37°C. PLA probe hybridization and ligation was performed using ligation solution for 30 min at 37°C. Polymerase solution was then added to the samples for 120 min at 37°C. The samples were mounted and examined using a light microscope.

### Protein–protein interaction networks

To identify common protein interactors between SNCA, MOBP and HIP1, we constructed a protein–protein interaction network using the Ingenuity Pathway Analysis software—IPA (QIAGEN Inc.; https://www.qiagenbioinformatics.com/products/ingenuity‐pathway‐analysis).^21^ SNCA, MOBP and HIP1 were used as seed proteins to construct the network, and only direct interactions and datasets with experimental evidence were considered.

## RESULTS

### Expression of *MOBP* and *HIP1* transcripts correlates with DNA methylation levels in MSA

**FIGURE 1:**
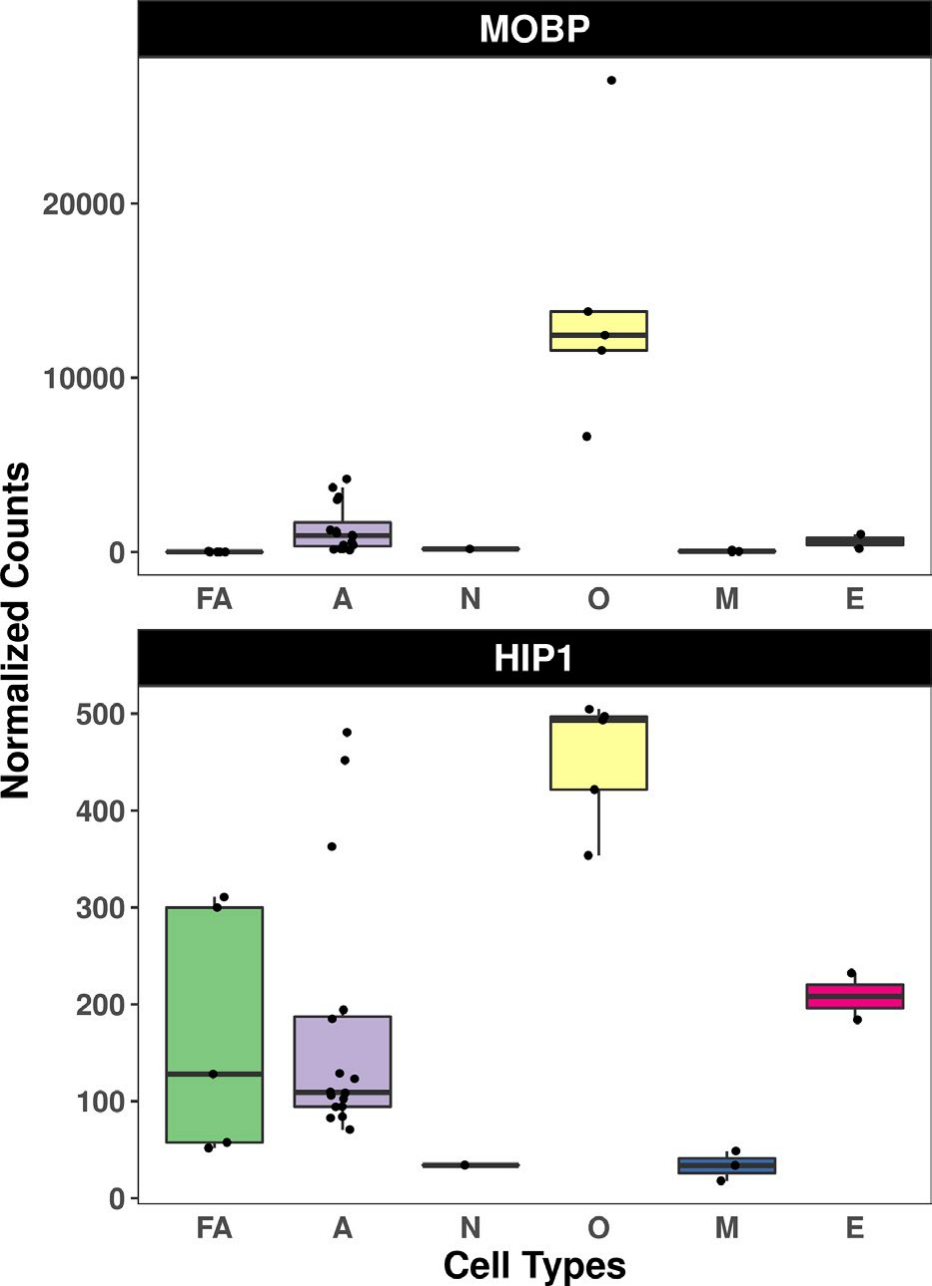
Boxplots showing gene expression levels for *MOBP* and *HIP1* across major brain cell types in healthy control brains. A, astrocytes; E, endothelial cells; FA, foetal astrocytes; M, microglia; N, neurons; O, oligodendrocytes

Our recent study revealed that *MOBP* and *HIP1* are among the most differentially methylated loci in MSA cerebellar white matter.^12^ To infer the mechanistic relevance of these DNA methylation changes, we assessed mRNA expression levels of these two loci. Our previous analyses of RNAseq data obtained from MSA cerebellar white matter and microdissected oligodendrocytes revealed no significant changes for *HIP1*. However, *MOBP* mRNA was significantly downregulated in MSA cases with a cerebellar phenotype as well as in MSA oligodendrocytes when compared to controls.^18^ As oligodendrocytes are the main focus of pathology in MSA, we investigated the underlying importance of *MOBP* and *HIP1* in the physiology of oligodendrocytes by analysing cell type‐specific RNAseq data from healthy controls (raw data from 19). We show that mRNA expression levels of both *MOBP* and *HIP1* are elevated in oligodendrocytes compared to other major brain cell types (Figure 1), supporting the hypothesis that they play an essential role in this cell type.

**FIGURE 2:**
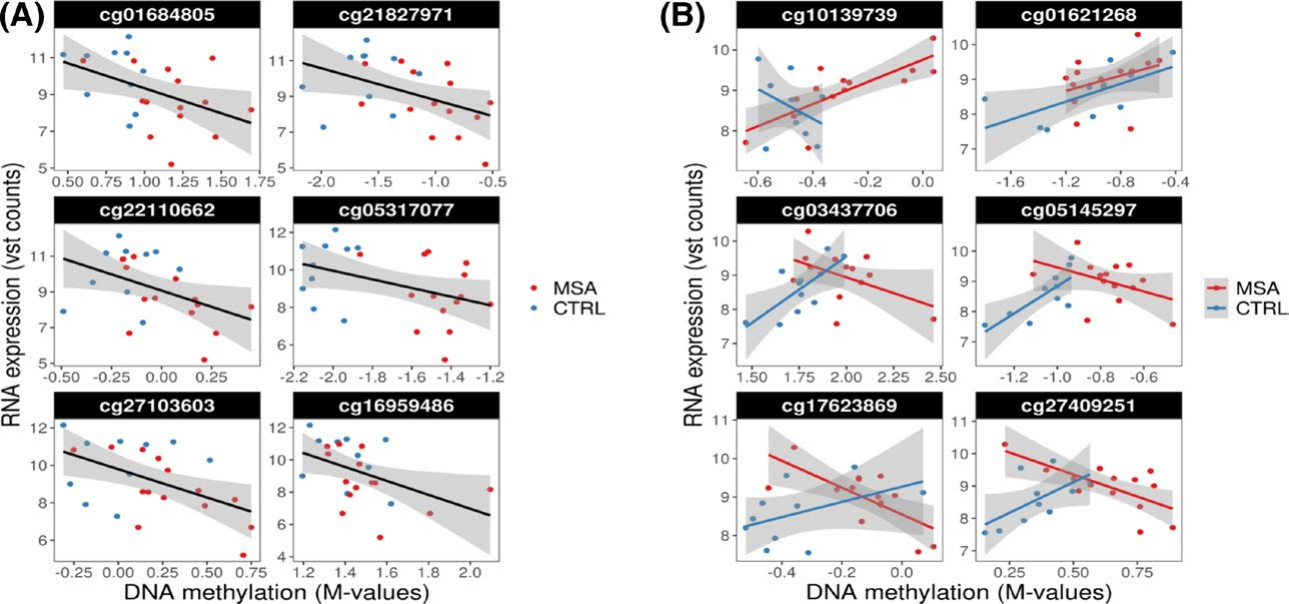
Scatterplots illustrating CpGs with significant DNA methylation‐expression correlations. (A) Six CpGs in *MOBP* with significant DNA methylation‐expression correlations (MSA + CTRL, *r* ≤ −0.411, *p* < 0.05). (B) Six CpGs in *HIP1* with significant DNA methylation‐expression correlations overall or in either MSA or healthy controls individually, most of which highlighting opposite correlation signals between MSA and healthy controls. CTRL, healthy controls; MSA, multiple system atrophy

To gain insights into the regulatory effect of DNA methylation on gene expression of *MOBP* and *HIP1*, we performed DNA methylation‐gene expression correlations in a subset of the MSA cases (*N* = 14) and controls (*N* = 10) from the RNAseq study^18^ with overlapping DNA methylation data.^12^ We observed a trend towards downregulation of *MOBP* in MSA (log2 FC = −1.480; adj. *p* = 0.343) with the same magnitude and direction of effect as the complete RNAseq dataset, although not reaching statistical significance likely due to the lack of statistical power in this subset of samples. As was observed in the complete RNAseq dataset, *HIP1* showed no significant differences in expression levels between MSA and controls (log2 FC = 0.322; adj. *p* = 0.751). Overall, for *MOBP,* we observed that DNA methylation levels in six CpGs (4/6 mapping to the promoter) were negatively correlated with *MOBP* mRNA levels (*r* ≤ −0.411, *p* < 0.05; Table 1; Figure 2), while for *HIP1* four CpGs mapping to the body of the gene were positively correlated with *HIP1* mRNA levels (*r* ≥ 0.437, *p* < 0.05; Table 1). This is in line with the widely accepted idea that the methylation status of the promoter exerts a repressive effect on proximal gene expression,^22^, ^23^ while CpGs inside gene bodies are more positively correlated with gene expression than promoter‐associated CpGs.^24^ For all six significant CpGs in *MOBP*, the correlation coefficients were stronger in MSA than in controls (Table 1), further supporting the idea that *MOBP* downregulation in MSA is driven by DNA methylation changes. While for three of the four *HIP1* CpGs, the correlation coefficients were stronger in the control group (Table 1). Additional CpGs in *HIP1* showed significant DNA methylation‐gene expression correlations in either MSA or controls separately (Table 1). For CpG sites in *HIP1*, we observed a total of 10.3% (7/68 CpGs) showing an opposite correlation signal between MSA and healthy controls (*r* ≥ |0.40|, e.g. cg03437706 MSA *r* = −0.48 vs. control *r* = 0.72; and cg27409251 MSA *r* = −0.69 vs. control *r* = 0.61; Figure 2; Table S2). To test the significance of this finding, we computed the correlation coefficients in MSA and healthy controls in 1000 random genes and found that the number of genes with the number of opposite correlations equal or larger than the 10.3% of CpG sites was much lower than the 5% cut‐off (*p* = 0.019), showing that the observations for *HIP1* are not likely to be due to chance. This suggests that *HIP1* methylation‐dependent regulatory activity is altered in MSA, implying the differential activity of other factors breaking the expected relationship between both variables.

**TABLE 1:** CpGs in *MOBP* and *HIP1* with significant DNA methylation‐gene expression correlations in cerebellar white matter of MSA cases and/or controls

Gene	CpG	Chr	Mapinfo	Feature‐CGI	MSA + CTRL		MSA		CTRL	
					*r*	*p*‐value	*r*	*p*‐value	*r*	*p*‐value
MOBP	cg27103603	3	39544721	Body‐shore	−0.513	**1.0E‐02**	−0.716	**4.0E‐03**	0.125	7.3E‐01
MOBP	cg16959486	3	39563230	Body‐opensea	−0.452	**2.6E‐02**	−0.405	1.5E‐01	−0.360	3.1E‐01
MOBP	cg01684805	3	39508348	TSS1500‐opensea	−0.451	**2.7E‐02**	−0.291	3.1E‐01	−0.266	4.6E‐01
MOBP	cg21827971	3	39542480	TSS1500‐shore	−0.440	**3.1E‐02**	−0.509	6.3E‐02	0.225	5.3E‐01
MOBP	cg22110662	3	39542841	TSS1500‐shore	−0.435	**3.4E‐02**	−0.594	**2.5E‐02**	0.281	4.3E‐01
MOBP	cg05317077	3	39542991	TSS1500‐shore	−0.411	**4.6E‐02**	−0.264	3.6E‐01	0.190	6.0E‐01
HIP1	cg10139739	7	75280150	Body‐opensea	**0.539**	**6.6E‐03**	**0.782**	**9.4E‐04**	−0.368	2.9E‐01
HIP1	cg14796107	7	75192547	Body‐shelf	**0.532**	**7.5E‐03**	0.319	2.7E‐01	**0.719**	**1.9E‐02**
HIP1	cg01621268	7	75272341	Body‐shelf	**0.529**	**7.8E‐03**	0.356	2.1E‐01	**0.646**	**4.4E‐02**
HIP1	cg00361176	7	75260791	Body‐opensea	**0.437**	**3.3E‐02**	−0.042	8.9E‐01	**0.587**	7.4E‐02
HIP1	cg03370878	7	75185075	Body‐shelf	−0.327	1.2E‐01	−0.745	**2.2E‐03**	0.000	1.0E+00
HIP1	cg11778783	7	75276901	Body‐opensea	−0.036	8.7E‐01	−0.718	**3.8E‐03**	0.074	8.4E‐01
HIP1	cg17623869	7	75251045	Body‐opensea	0.047	8.3E‐01	−0.703	**5.0E‐03**	**0.463**	1.8E‐01
HIP1	cg11416840	7	75250896	Body‐opensea	−0.303	1.5E‐01	−0.689	**6.4E‐03**	−0.078	8.3E‐01
HIP1	cg27409251	7	75223159	Body‐opensea	0.008	9.7E‐01	−0.686	**6.8E‐03**	**0.610**	6.1E‐02
HIP1	cg02229461	7	75185674	Body‐shelf	0.031	8.9E‐01	−0.614	**1.9E‐02**	0.365	3.0E‐01
HIP1	cg05421036	7	75202434	Body‐opensea	0.067	7.6E‐01	−0.541	**4.6E‐02**	**0.629**	5.1E‐02
HIP1	cg01120324	7	75249065	Body‐opensea	0.262	2.2E‐01	0.229	4.3E‐01	**0.852**	**1.8E‐03**
HIP1	cg05145297	7	75264568	Body‐shelf	0.229	2.8E‐01	−0.420	1.4E‐01	**0.772**	**8.9E‐03**
HIP1	cg26596975	7	75189250	Body‐island	0.192	3.7E‐01	0.018	9.5E‐01	**0.732**	**1.6E‐02**
HIP1	cg03437706	7	75266692	Body‐shore	0.111	6.0E‐01	−0.486	7.8E‐02	**0.722**	**1.8E‐02**
HIP1	cg14706940	7	75205521	Body‐opensea	0.346	9.8E‐02	−0.014	9.6E‐01	**0.672**	**3.3E‐02**
HIP1	cg02713883	7	75188876	Body‐shore	0.253	2.3E‐01	0.240	4.1E‐01	**0.660**	**3.8E‐02**

Note

Significant *p*‐values (*p* < 0.05) are highlighted in bold; correlation coefficients *r* ≥ 0.4 are underlined and in bold; correlation coefficients *r* ≤ −0.4 are underlined.

Abbreviations: CGI, CpG Island; Chr, chromosome; CTRL, healthy controls; Mapinfo, genomic location; MSA, multiple system atrophy.

### MOBP and HIP1 protein levels did not differ between MSA and controls but expression patterns vary with disease stage

**FIGURE 3:**
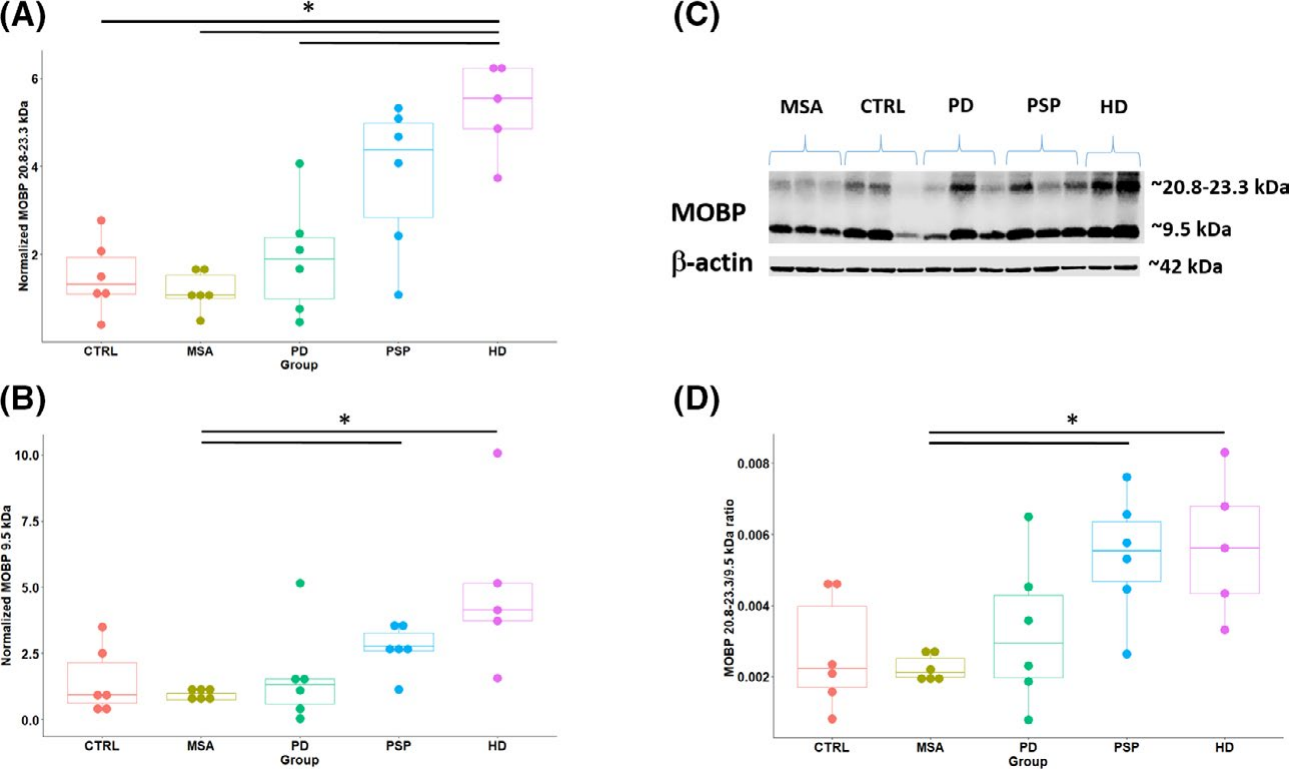
Western blot detection of MOBP in cerebellar white matter lysates (*N* = 29). Immunoblots were labelled with anti‐MOBP (Atlas Antibodies HPA035152, 1:250), anti‐β‐actin (Sigma A1978, 1:5000) and IRDye secondary antibodies (LiCor), and scanned on a LiCor Odyssey Fc (C). Intensities for the 20.8–23.3 kDa (isoforms *b* and *a*) and 9.5 kDa (isoform *c*) bands, respectively, were derived from raw scan data, analysed on Image Studio (LiCor), and normalized against β‐actin band for each sample as housekeeping gene (A, B). Analysis of the ratio between levels of 20.8–23.3 kDa (isoforms *b* and *a*) over 9.5 kDa (isoform *c*) bands (D). *Horizontal black bars indicate significant differences (adjusted *p* < 0.05 using Wilcoxon rank‐sum test with Benjamini–Hochberg adjustment for multiple testing). CTRL, healthy controls; MSA, multiple system atrophy; PD, Parkinson's disease; PSP, progressive supranuclear palsy; HD, Huntington's disease.

**FIGURE 4:**
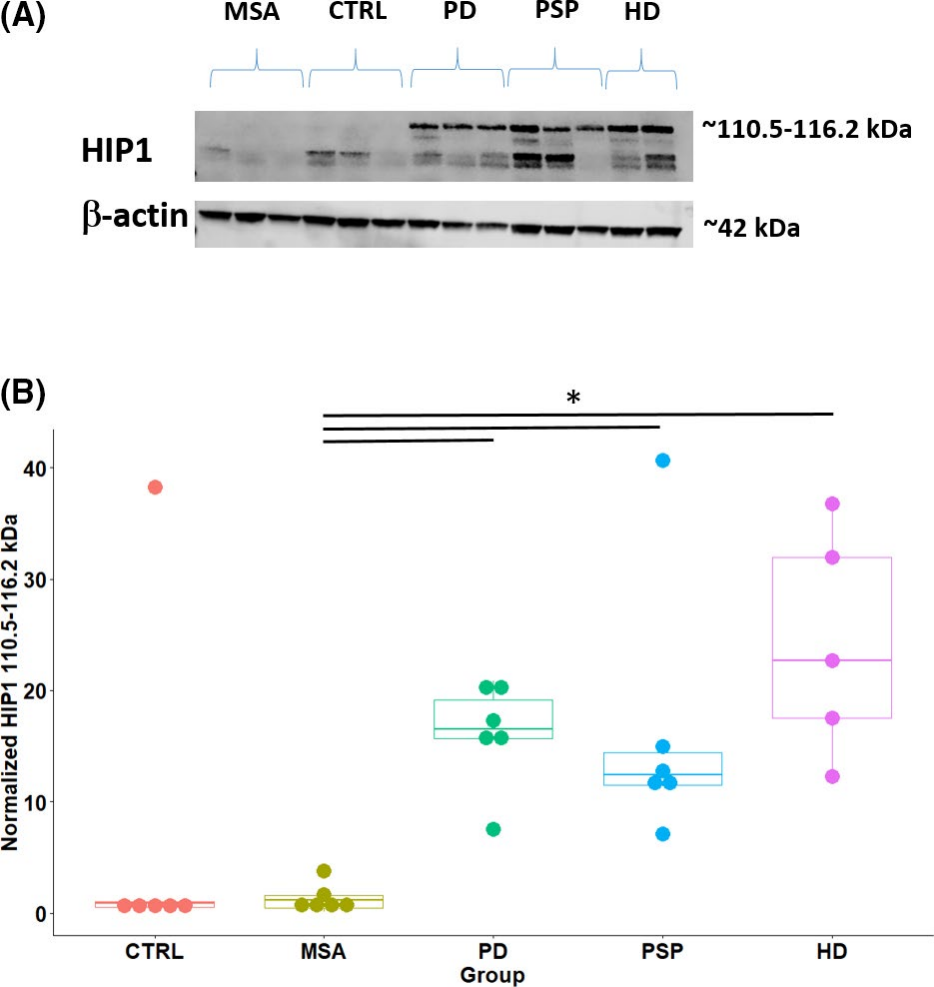
Western blot detection of HIP1 in cerebellar white matter lysates (*N* = 29). Immunoblots were labelled with anti‐HIP1 (Abcam ab181238, 1:2000), anti‐β‐actin (Sigma A1978, 1:5000) and IRDye secondary antibodies (LiCor), and scanned on a LiCor Odyssey Fc (A). Band intensities were derived from raw scan data and analysed on Image Studio (LiCor). HIP1 band intensities normalized against β‐actin as housekeeping gene (B). *Horizontal black bars indicate significant differences (adjusted *p* < 0.05 using Wilcoxon rank‐sum test with Benjamini–Hochberg adjustment for multiple testing). CTRL, healthy controls; HD, Huntington's disease; MSA, multiple system atrophy; PD, Parkinson's disease; PSP, progressive supranuclear palsy

We then investigated cerebellar white matter soluble protein levels for both MOBP and HIP1 in MSA (*N* = 6) and controls (*N* = 6) by western blotting. Alternative splicing variants have been described for *MOBP* and *HIP1*, and for both we observed several bands likely to correspond to distinct protein isoforms (Figure 3 and 4).

MOBP has three known isoforms (RefSeq: isoform *a* [NP_001265251], isoform *b* [NP_001265252] and isoform *c* [NP_891980]) with molecular weights of about 23.3, 20.8 and 9.5 kDa, respectively. As expected, in cerebellar white matter samples, we mainly detected the dominant ~9.5 kDa MOBP isoform *c*. The two larger isoforms, *a* and *b*, are also present but less abundant (fainter band at ~20.8–23.3 kDa) (Figure 3; Figure S1). We did not detect significant changes in soluble MOBP protein levels when compared to controls. The 9.5 kDa isoform *c* or isoforms *b* and *a* (20.8 and 23.3 kDa) remained unaltered, and similarly total MOBP or the ratio between the two ((*b* + *a*)/*c*) was unchanged between MSA and controls. These results contrast with the finding that *MOBP* mRNA was found downregulated in MSA, suggesting the involvement of post‐transcriptional mechanisms regulating MOBP protein levels in MSA.

For HIP1, we also observed multiple peptides (Figure 4; Figure S2), including a doublet just over 62 kDa and a more distinct band at around 115 kDa. The six known HIP1 isoforms (RefSeq: NP_005329.3, NP_001230127.1, NP_001369373.1, NP_001369374.1, XP_011514418.1, XP_016867588.1) have calculated molecular weights between 110.5 and 116.2 kDa, one or more of which likely corresponding to the highest molecular weight band we observed (Figure 4). For the 110.5–116.2 kDa isoforms, all controls, with the exception of one outlier, showed comparable HIP1 levels to those observed in MSA, and no significant differences were detected between MSA and controls. In fact, the 110.5–116.2 kDa isoforms are barely detectable in both MSA and controls. The bands corresponding to smaller molecular weight peptides that were consistently observed may represent additional HIP1 isoforms or HIP1 degradation products but were not quantified (Figure 4; Figure S2).

We also investigated the levels MOBP and HIP1 proteins in the occipital lobe white matter, which is usually minimally affected in MSA and should represent an earlier stage of the disease progression. Although the median levels of expression of MOBP (9.5 and 20.8–23.3 kDa) and HIP1 were higher in MSA when compared to controls, similar to what we observed in the cerebellar white matter, no significant differences were found between MSA and healthy controls (Figures S3 and S4).

It is of note that while in MSA cerebellar white matter we observed a very strong correlation between the levels of HIP1 protein and MOBP 20.8–23.3 kDa isoforms (*r* = 0.90, *p* = 0.01), in the occipital lobe white matter this co‐expression pattern was not observed (*r* = −0.49, *p* > 0.05; Figure S5). Similar to the latter, in healthy controls such a co‐expression pattern between HIP1 and MOBP 20.8–23.3 kDa was not found (Figure S5).

To get further insights into the involvement of these proteins in MSA pathogenesis, we investigated the relationship between MOBP and HIP1 protein levels and MSA disease traits. We found that, in the occipital white matter, the levels of MOBP 20.8–23.3 kDa are inversely correlated with the MSA disease duration (*r* = −0.93, *p* = 0.008), i.e., the levels of these MOBP isoforms are decreased in patients with longer disease duration, while HIP1 shows the opposite trend (*r* = 0.75, *p* = 0.084; Figure S6). These findings point towards perturbation of the levels of MOBP 20.8–23.3 kDa and HIP1 proteins in opposite directions during MSA disease progression in a very mildly affected brain region (occipital lobe), which should represent an earlier stage of the disease pathogenesis. However, at later stages of the disease process, as represented by the observations in the cerebellar white matter (where no significant correlations were found with disease duration), the levels of these proteins converge and both proteins seem to be co‐expressed (Figure S5).

### MOBP and HIP1 protein levels differed between MSA and other neurodegenerative diseases

We also investigated the levels HIP1 and MOBP proteins in other neurodegenerative disorders, including PD (*N* = 6) as another synucleinopathy, PSP (*N* = 6) as a disease in which variants in *MOBP* are a genetic risk factor and HD (*N* = 5) as a disease in which the mutant protein HTT is known to be a HIP1 interactor. For MOBP (Figure 3; Figure S1), the predominant 9.5 kDa isoform *c* levels were lower in MSA compared to PSP (adj. *p* = 0.041) and HD (adj. *p* = 0.041). Levels of the larger MOBP isoforms *b* and *a* (20.8 and 23.3 kDa) were lower in MSA compared to HD (adj. *p* = 0.029), while for total MOBP (isoforms *a* + *b* + *c*), protein levels were additionally lower in MSA compared to PSP (adj. *p* = 0.041). Controls and PD also displayed lower levels of MOBP isoforms *b* and *a* as well as total MOBP when compared to HD (adj. *p* < 0.05). The ratio of isoforms (*b* + *a*)/*c* significantly distinguishes MSA from PSP and HD (adj. *p* = 0.022). For HIP1 (Figure 4; Figure S2), 110.5–116.2 kDa isoforms levels were significantly lower in MSA compared to PD (adj. *p* = 0.011), PSP (adj. *p* = 0.011) and HD (adj. *p* = 0.014). No significant differences were detected when comparing the other diseases with controls, despite all controls, with the exception of one outlier, presenting HIP1 levels comparable to MSA and much lower than PD, PSP and HD.

### MOBP and HIP1 are mislocalized in MSA cerebellar white matter and interact with SNCA in GCIs

**FIGURE 5:**
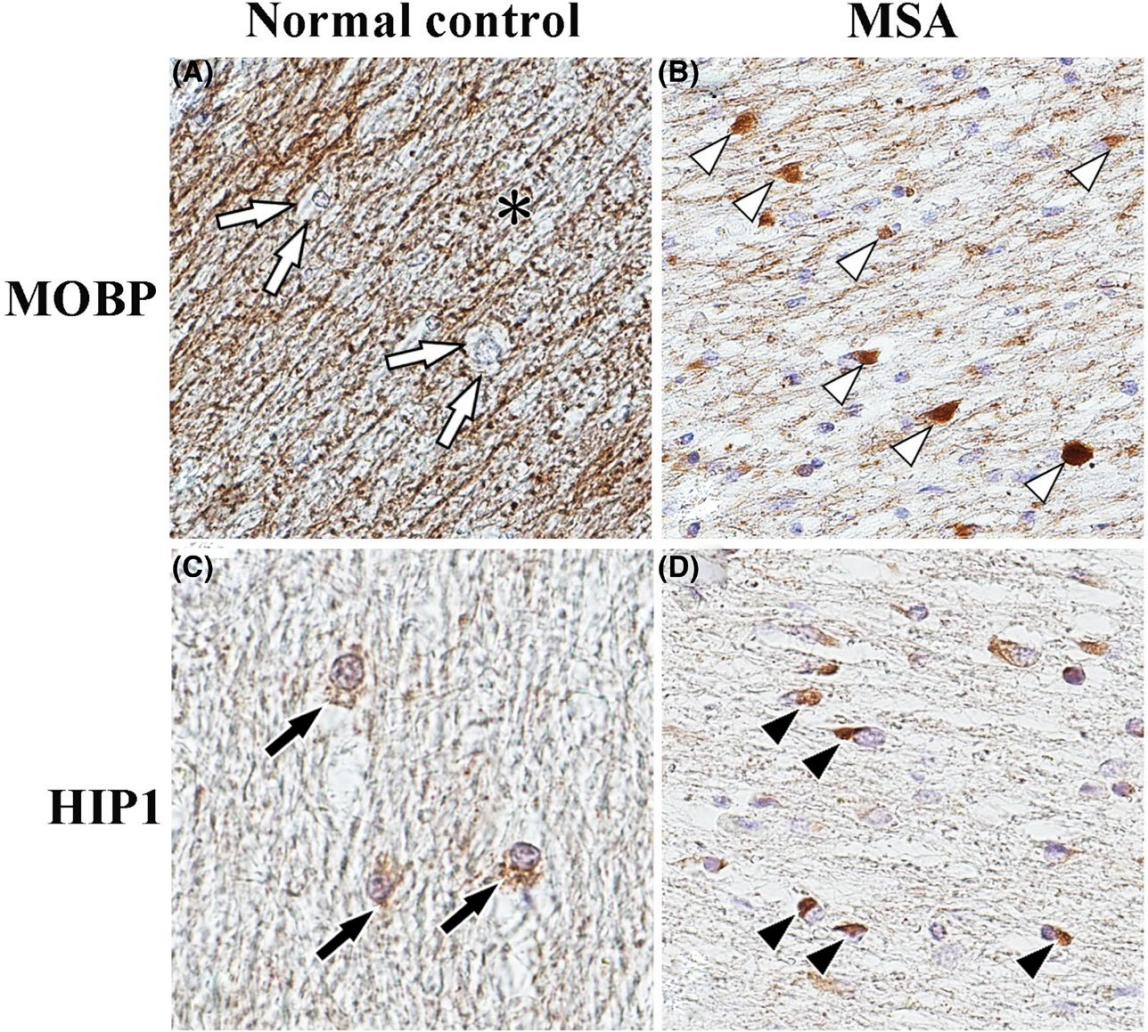
Immunoreactivity of MOBP and HIP in healthy controls (*N* = 5) and MSA patients (*N* = 5). In healthy controls, myelin sheaths were immunolabelled with anti MOBP antibody (asterisk), whereas the cytoplasm of oligodendroglia was immunonegative for MOBP (white arrows) (A). In patients with MSA, glial cytoplasmic inclusions (GCIs) were strongly immunopositive for MOBP (white arrowheads) (B). In healthy controls, weak, granular immunoreactivity for HIP1 was seen in the cytoplasm of oligodendroglia (black arrows) (C). In contrast, HIP1 intense immunoreactivity was observed in GCIs (black arrowheads) (D)

Using immunohistochemistry, we investigated the localization of MOBP and HIP1 in cerebellar white matter. For MOBP, myelin sheaths were immunopositive and the cytoplasm of oligodendroglia was immunonegative in healthy controls (*N* = 5), while in patients with MSA (*N* = 5), GCIs were strongly immunopositive for MOBP (Figure 5). For HIP1, a weak and granular immunoreactivity was seen in the cytoplasm of oligodendroglia of healthy controls, while an intense immunoreactivity was observed in GCIs of patients with MSA (Figure 5).

**FIGURE 6:**
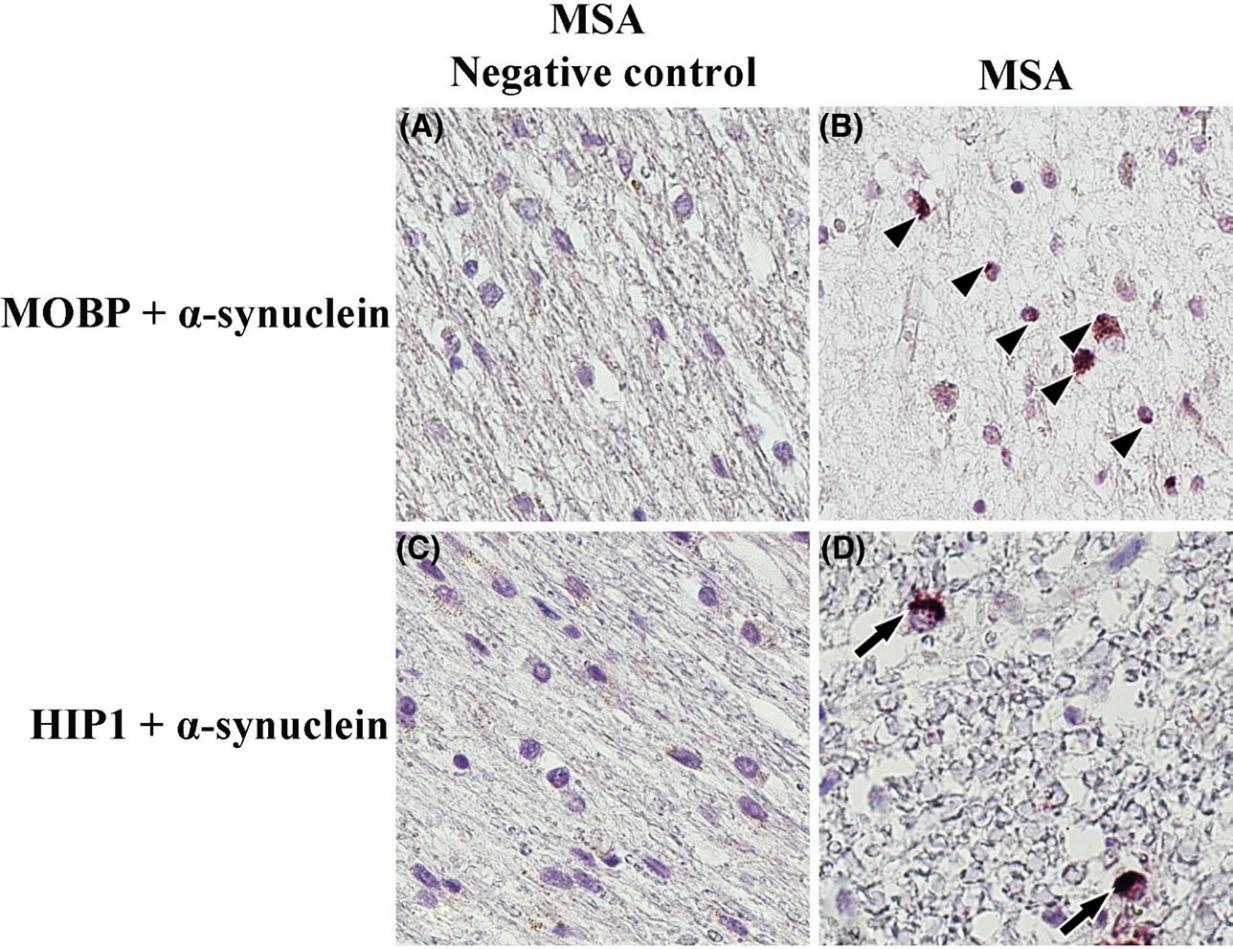
Protein–protein interactions of alpha‐synuclein and MOBP or HIP1 in cerebellar white matter of patients with MSA (*N* = 2). Proximity ligation assay was performed to investigate protein–protein interactions of α‐synuclein and MOBP or HIP1. In the absence of primary antibodies, no signals were detected in the cytoplasm of oligodendroglia (A, C). In patients with MSA, discrete signals in GCIs were observed (B, D), indicating the protein–protein interactions of α‐synuclein and MOBP (B; black arrowheads) and HIP1 (D; black arrows).

To investigate the co‐localization of MOBP or HIP1 with SNCA in GCIs and the interaction of these proteins with SNCA, we performed PLAs in cerebellar tissue of MSA patients (*N* = 2). In the absence of primary antibodies, no signals were detected (Figures 6a,c), whereas, in the presence of primary antibodies for MOBP and SNCA, binding of MOBP and SNCA was demonstrated in GCIs (Figure 6b). Similarly, in the presence of primary antibodies, binding of HIP1 and SNCA was demonstrated in GCIs (Figure 6d). No binding of MOBP or HIP1 and SNCA was observed at synaptic terminals (data not shown).

We then investigated the specificity of the mislocalization of MOBP and HIP1 into GCIs in MSA by comparing with the disease controls (PD, PSP and HD). Both Lewy bodies in PD and intranuclear inclusions in HD were immunonegative for both MOBP and HIP1, while in PSP we observed that occasional inclusions were immunopositive for HIP1 (e.g. coiled bodies in frontal cortex oligodendrocytes) but not for MOBP (Figure S7).

### Shared protein–protein interactors across SNCA, MOBP and HIP1

**FIGURE 7:**
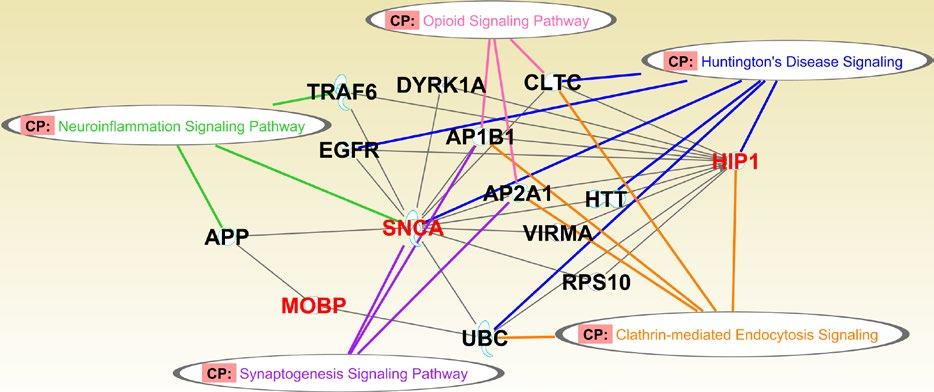
Shared protein interactors for α‐synuclein (SNCA), HIP1 and MOBP identified through the construction of a protein–protein interaction network using IPA. APP, amyloid precursor protein; AP1B1, adaptor‐related protein complex 1 subunit beta 1; AP2A1, adaptor‐related protein complex 2 subunit alpha 1; CLTC, clathrin heavy chain; CP, canonical pathways; DYRK1A, dual specificity tyrosine phosphorylation‐regulated kinase 1A; EGFR, epidermal growth factor receptor; HTT, huntingtin; RPS10, ribosomal protein S10; TRAF6, TNF receptor‐associated factor 6; UBC, ubiquitin C; VIRMA, vir like m6A methyltransferase associated

Based on the SNCA, MOBP and HIP1 protein–protein interaction network constructed with IPA^21^ (Table S3), we identified ubiquitin C (UBC) as a common interactor for SNCA, MOBP and HIP1 (Figure 7). Additional interactors of at least two of these three proteins were also observed (Figure 7). It is of note that neither MOBP nor HIP1 have been previously reported to interact with SNCA. The overlay of this protein–protein interaction network with canonical pathways revealed HD Signalling and Clathrin‐mediated Endocytosis Signalling as the top pathways with the higher number of interactor members (Figure 7).

## DISCUSSION

In our previous MSA epigenome‐wide study, we reported that *MOBP* and *HIP1* were consistently differentially methylated in MSA compared to controls. Another study investigating common DNA methylation changes across several neurodegenerative diseases^25^ has suggested shared changes in *HIP1* (Alzheimer's disease and Down Syndrome) and *MOBP* (PD and dementia with Lewy bodies), supporting the notion that DNA methylation changes in these genes can have a broader relevance for neurodegenerative diseases. Furthermore, as these two genes are more highly expressed in oligodendrocytes than in other major brain cell types, they are likely to play an essential role in this cell type, which is where GCIs occur in MSA. This prompted us to investigate functional downstream alterations in mRNA expression and its correlation with DNA methylation levels as well as changes in protein expression and localization in MSA, leading to the identification of MOBP and HIP1 as new constituents of GCIs.

The *MOBP* gene encodes the myelin associated oligodendrocyte basic protein (MOBP), which is the third‐most abundant protein in central nervous system (CNS) myelin.^26^ The observed downregulation of *MOBP* mRNA expression levels in MSA cases was correlated with higher levels of DNA methylation in CpGs mapping to the *MOBP* promoter, suggesting that the variation in the mRNA expression levels observed in MSA may be driven by changes in DNA methylation. Genetic variants in the *MOBP* gene have been previously associated with neurodegenerative diseases, including PSP and corticobasal degeneration, in which genetic variants in this gene associate with the disease risk.^15^, ^16^ Although upregulation of *MOBP* expression has been observed in PSP, a study investigating DNA methylation‐gene expression correlations in PSP brain tissue failed to find significant correlations for *MOBP*,^27^ suggesting the involvement of different mechanisms in different diseases.

The *HIP1* gene encodes a cytosolic protein (huntingtin interacting protein 1—HIP1), which is ubiquitously expressed and highly enriched in human and mouse brain tissue.^17^ We found no consistent mRNA expression changes for *HIP1* in MSA. However, we observed a complex relationship between *HIP1* DNA methylation and gene expression levels, which differed between MSA and controls, suggesting that *HIP1* methylation‐dependent regulatory activity might be altered in MSA. Epigenetic remodelling, with substantial changes in DNA methylation‐gene expression correlation between normal and disease tissue, has been reported in cancer.^28^ This implies differential activity of other factors breaking the expected relationship between DNA methylation and gene expression.^28^

The presence of GCIs in oligodendrocytes, the primary pathological hallmark of MSA, and myelin dysfunction have been shown to precede neurodegeneration in MSA.^29^, ^30^ The relevance of GCIs for MSA pathogenesis is emphasized by the fact that the number of GCIs correlates with disease duration and the severity of neurodegeneration.^4^ Fibrillary SNCA is the main component of GCIs and is thought to play a role in GCI formation.^31^ However, it is not clear whether SNCA deposition in GCIs originates from increased gene expression in oligodendrocytes and/or from the uptake from neurons or the extracellular space. Ubiquitin as well as cytoskeletal proteins (e.g. p25α/TPPP, non‐phosphorylated tau), myelin‐related proteins (e.g. myelin basic protein [MBP] and MOG) and many other proteins have been shown to be part of GCIs.^30^, ^32^ Following up relevant loci from our MSA DNA methylation study^12^ led us to identify the mislocalization of HIP1 and MOBP into GCIs in MSA, where they seem to interact with SNCA.

We found MOBP isoforms with multiple molecular weights, but no significant differences were observed in the levels of soluble MOBP protein between MSA and controls. This finding contrasts with the downregulation of *MOBP* mRNA observed in MSA, and points towards post‐transcriptional mechanisms regulating MOBP protein levels in MSA. The exact function of MOBP remains unclear, but it has been suggested that it participates in myelin stabilization by connecting the myelin to a membrane‐associated signalling complex linked to the cytoskeleton.^26^ Myelin dysfunction has been described as an early event in MSA,^30^ and our results suggest that, in addition to other myelin‐related constituents of GCIs (e.g. MBP), MOBP may contribute to such dysfunction. Like *MBP* mRNA, *MOBP* mRNA is thought to be dynamically translocated from the oligodendrocyte cell body to the processes and translated locally during myelination.^26^ Members of the QKI, KH domain containing RNA binding (QKI) family of proteins, which play a crucial role in CNS RNA metabolism, have been reported as being involved in such translocation.^26^, ^33^ Interestingly, we have previously shown that, in MSA cerebellar white matter, *MOBP* as well as *HIP1*, *TPPP*, *MBP* and other myelin‐related genes are co‐expressed with *QKI*. This co‐expression cluster, which has the *QKI* gene as the hub gene (the highest interconnected gene), is downregulated in MSA and is enriched for several biological processes, including myelination.^18^ Furthermore, in QKI‐depleted oligodendrocytes, there is downregulation of myelin‐related genes (including *MOBP*),^33^ significantly overlapping with genes we found in the MSA‐associated *QKI* co‐expression cluster.^18^ It is also of note that, in our previous study, *QKI* was part of co‐methylation signatures significantly hypermethylated in MSA when compared to healthy controls.^12^ We speculate that *MOBP* mRNA translocation to the myelin might be affected in MSA via downregulation of *QKI*, contributing to the myelin dysfunction observed early in MSA. This may in turn lead to MOBP translation in the oligodendrocyte cell body, and facilitate its subsequent sequestration into GCIs, which would prevent its degradation, and explain the absence of changes in MOBP protein levels between MSA and healthy controls, despite the observed *MOBP* mRNA downregulation in MSA cerebellar white matter. Our findings in the occipital lobe white matter, which represent an earlier stage of the MSA pathological changes, suggest that indeed the MOBP levels decrease in an isoform‐specific way as the disease progresses. Little is known about the function of different MOBP isoforms. These findings warrant further investigation in future studies.

For HIP1, in line with our observations for the mRNA, we found no significant differences in the soluble protein levels between MSA and healthy controls in cerebellar white matter. Similar findings were observed in occipital lobe white matter. However, although one should be cautious while interpreting the results from a relatively small sample size, in this mildly affected brain region in MSA, the median levels of HIP1 protein seem slightly higher in MSA than in controls and its levels seem to increase with the progression of the disease. Although little is known of the function of HIP1 protein, it has been shown to have a role in the clathrin‐mediated endocytosis, which regulates several signalling pathways, receptor trafficking and cytoskeleton dynamics.^34^, ^35^ It is interesting to note that HIP1(−/−) mice develop a neurological phenotype by 3 months of age, including tremor and a gait ataxia secondary to a rigid thoracolumbar kyphosis accompanied by decreased assembly of endocytic protein complexes on liposomal membranes.^36^ Our protein–protein network analysis revealed common interactors between HIP1 and SNCA, several of which are involved in clathrin‐mediated endocytosis signalling. Recent studies have implicated SNCA in clathrin assembly and in changes in clathrin‐mediated endocytosis.^37^, ^38^ We previously found significant downregulation of *SNCA* mRNA in MSA cerebellar white matter, and very low levels of *SNCA* mRNA in microdissected oligodendrocytes.^18^ These observations support the hypothesis that the accumulation of SNCA in oligodendrocytes in MSA is contributed to by SNCA uptake from neurons or from the extracellular environment through a clathrin‐dependent internalization mechanism,^30^, ^39^ a process in which HIP1 may be involved.

Our protein–protein interaction networks show EGFR (epidermal growth factor receptor) as a shared interactor between SNCA and HIP1. As EGFR signalling regulates oligodendrogenesis and remyelination,^40^ this could point to a possible link among HIP1, SNCA and alterations to myelination. Activated EGFR has been proposed in the pathophysiology of neurodegenerative diseases and neuroinflammation.^41^ Therapies targeting EGFR are standard in several cancers,^42^ and have also been proposed as promising inhibitors of neuroinflammation in the CNS neurodegenerative diseases.^41^ Recent studies support a role of DNA methylation in neuroinflammatory responses in MSA,^13^, ^43^ and an early crosstalk between neuroinflammation and oligodendrocytes containing GCIs leading to an immune response locally restricted to white matter regions has been reported in MSA.^44^ Whether this involves EGFR signalling remains unknown.

Our protein–protein network analysis also showed UBC sharing interactions across SNCA, MOBP and HIP1. The polyubiquitin precursors UBC and UBB are major determinants of the intracellular ubiquitin content under basal conditions and providers of the extra ubiquitin needed in stressful conditions.^45^, ^46^ Through our gene expression study,^18^ we showed that *UBB* is significantly downregulated in MSA cerebellar white matter, and identified is a trend for *UBC* upregulation in microdissected oligodendrocytes. Ubiquitin is a known component of GCIs,^30^, ^32^ but whether UBC is involved in the pathogenesis of MSA remains to be explored. Our previous functional network analysis revealed a common transcriptional background between MSA and Alzheimer's disease, which among other genes included amyloid precursor protein (*APP*) (downregulated in MSA) and *DYRK1* (upregulated in MSA).^18^ It is interesting to note that APP is a shared interactor between SNCA and MOBP, and DYRK1 is a shared interactor between SNCA and HIP1.

The fact that (a) *EGFR*, *HTT* and *UBC* are among genes composing co‐methylation signatures associated with MSA, and within the same clusters as *SNCA* or *HIP1*,^12^ suggests related molecular functions among those genes, and (b) EGFR, HTT and UBC are experimentally validated protein interactors of SNCA, MOBP and/or HIP1, as seen in our protein–protein interaction network, further supports a functional role for DNA methylation changes in MSA pathogenesis.

From our comparisons across neurodegenerative diseases, we found that soluble MOBP protein levels in cerebellar white matter did not differ between the two synucleinopathies (MSA and PD). Furthermore, we found strong immunopositive staining for MOBP in MSA cerebellar GCIs but not in midbrain PD Lewy bodies, nor in frontal cortex PSP or HD inclusions. Our findings contrast with results from a recent study by Kon et al.,^47^ who reported negative immunoreactivity for MOBP in cerebellar MSA GCIs, but positive immunoreactivity for MOBP in a proportion of Lewy bodies in PD and dementia with Lewy bodies cases. Technical differences between these two studies may contribute to the observed discrepancy in the immunostaining. In the present study, MOBP protein levels did distinguish MSA from PSP and HD, two diseases of the tauopathy spectrum.^48^, ^49^ Although tau is a known component of GCIs in MSA, its phosphorylation state in GCIs differs from that seen in tauopathies.^30^, ^32^ In addition to showing immunopositivity in GCIs in MSA, HIP1 also positively stained a small proportion of proteinaceous inclusions in PSP (e.g. coiled bodies), while it was negative in PD and HD proteinaceous inclusions. HIP1 protein levels distinguished MSA from all the other neurodegenerative diseases investigated, being overexpressed in PD, PSP and HD when compared to MSA. Given that HIP1 is overexpressed in multiple cancer types, including oligodendrogliomas, it has been proposed as a tumour marker.^50^, ^51^ Our results suggest that HIP1 and, to some extent MOBP, may be candidate markers in neurodegenerative diseases.

Overall, by investigating loci that have shown significant DNA methylation alterations in MSA, this study identified MOBP and HIP1 as new constituents of GCIs and SNCA interactors, thus strengthening the potential role of these two loci in MSA pathogenesis. Given the previously reported functions for MOBP and HIP1, our results suggest myelin dysfunction and clathrin dependent endocytosis as important mechanisms in MSA, which warrants further investigation in future studies. Our findings also raise new candidate biomarkers and possible targets for therapeutic intervention.

## CONFLICT OF INTEREST

The authors declare that they have no conflict of interest.

AUTHOR CONTRIBUTION

Conceição Bettencourt contributed to the design of the study, experimental work, analysis and interpretation of data, and drafted the manuscript; Yasuo Miki contributed to the experimental work, and revised clinical and pathological data; Ignazio S. Piras and Rohan de Silva contributed to the analysis and interpretation of data; Sandrine C. Foti and Joshua S. Talboom contributed to the generation of the data; Thomas T. Warner and Tamas Revesz revised clinical and/or pathological data; Emmanuelle Viré, Robert Balazs and Tammaryn Lashley contributed for the design of the work, and interpretation of the data; Matt J. Huentelman and Janice L. Holton made substantial contributions to the conception and supervision of the work. All authors have critically revised the manuscript and approved the submitted version.

## ETHICAL APPROVAL

All tissue came from brains donated to the Queen Square Brain Bank for Neurological Disorders, where tissue is stored under a licence from the Human Tissue Authority. The brain donation programme and protocols have received ethical approval for donation and research by the NRES Committee London—Central (18/LO/0721).

### Peer Review

The peer review history for this article is available at https://publons.com/publon/10.1111/nan.12688.

## Supporting information

Fig S1‐S2

Fig S3‐S7

Table S1

Table S2

Table S3

## Data Availability

The data that support the findings of this study are available in the Supporting Information of this article, and from the corresponding author upon reasonable request.
