# Supplementary material for: MOBP and HIP1 in multiple system atrophy: New α‐synuclein partners in glial cytoplasmic inclusions implicated in the disease pathogenesis
Source: Neuropathol Appl Neurobiol. 2021 Jan 19;47(5):640–52. doi: 10.1111/nan.12688 (PMC8219819; doi:10.1111/nan.12688)
Supplement: Supplementary file 1 — Fig S1‐S2 [file NAN-47-640-s001.pdf]

Supplementary Figure 1: Original full length MOBP immunoblots related to Figure 4. Immunoblots were labelled with anti-MOBP (1:250; Atlas Antibodies HPA035152), anti-β-actin (1:5,000; Sigma A1978) and IRDye secondary antibodies (LiCor), and scanned on a LiCor Odyssey Fc. Samples were run in a total of 4 gels (**a-d**): **b** is a duplicate of **a**; **d** is a duplicate of **c**.

a

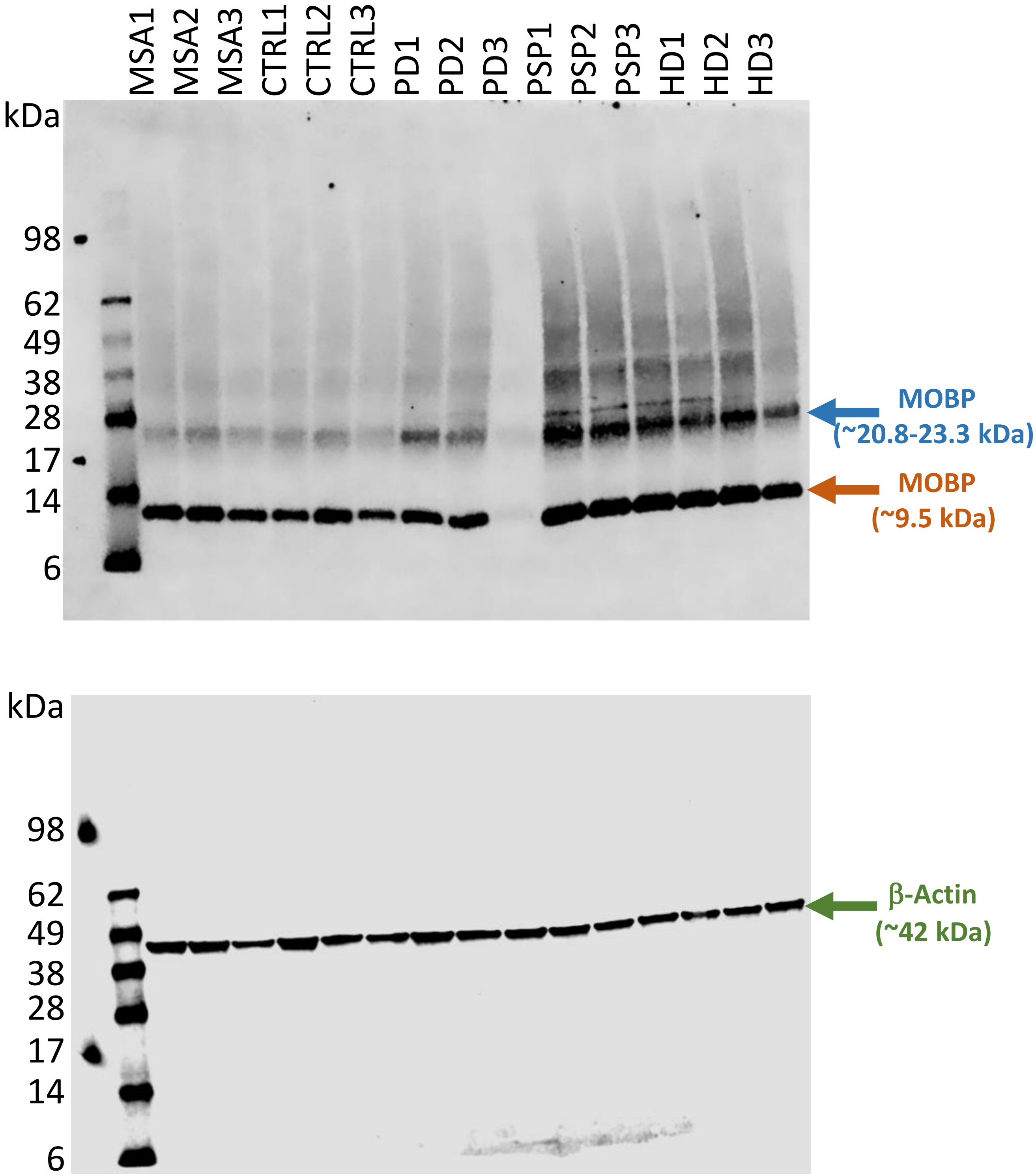

b

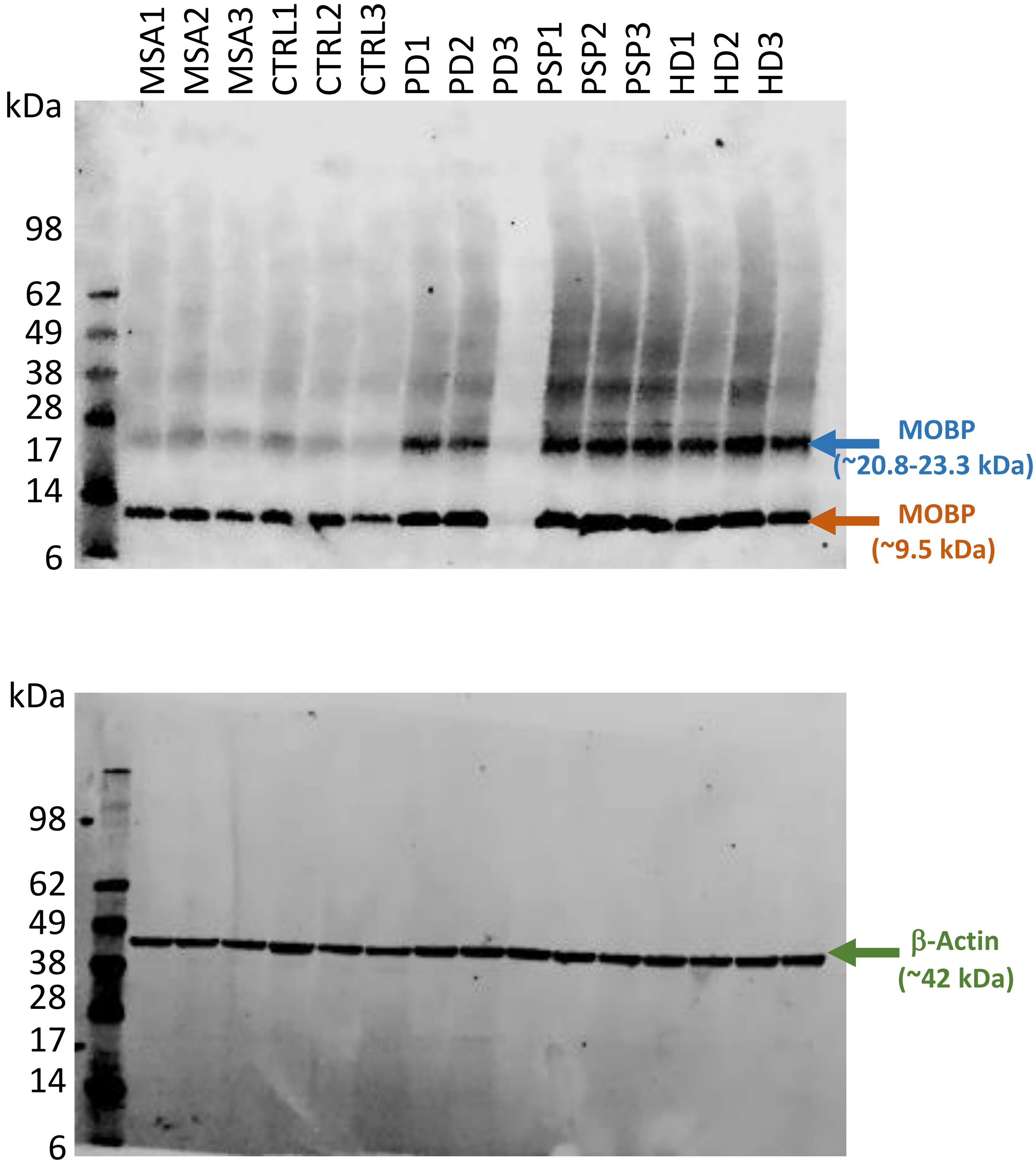

MSA1
MSA2
MSA3
CTRL1
CTRL2
CTRL3
PD1
PD2
PD3
PSP1
PSP2
PSP3
HD1
HD2
HD3
kDa
98
62
49
38
28
17
14
6
MOBP
(~20.8-23.3 kDa)
MOBP
(~9.5 kDa)
kDa
98
62
49
38
28
17
14
6
β-Actin
(~42 kDa)

c

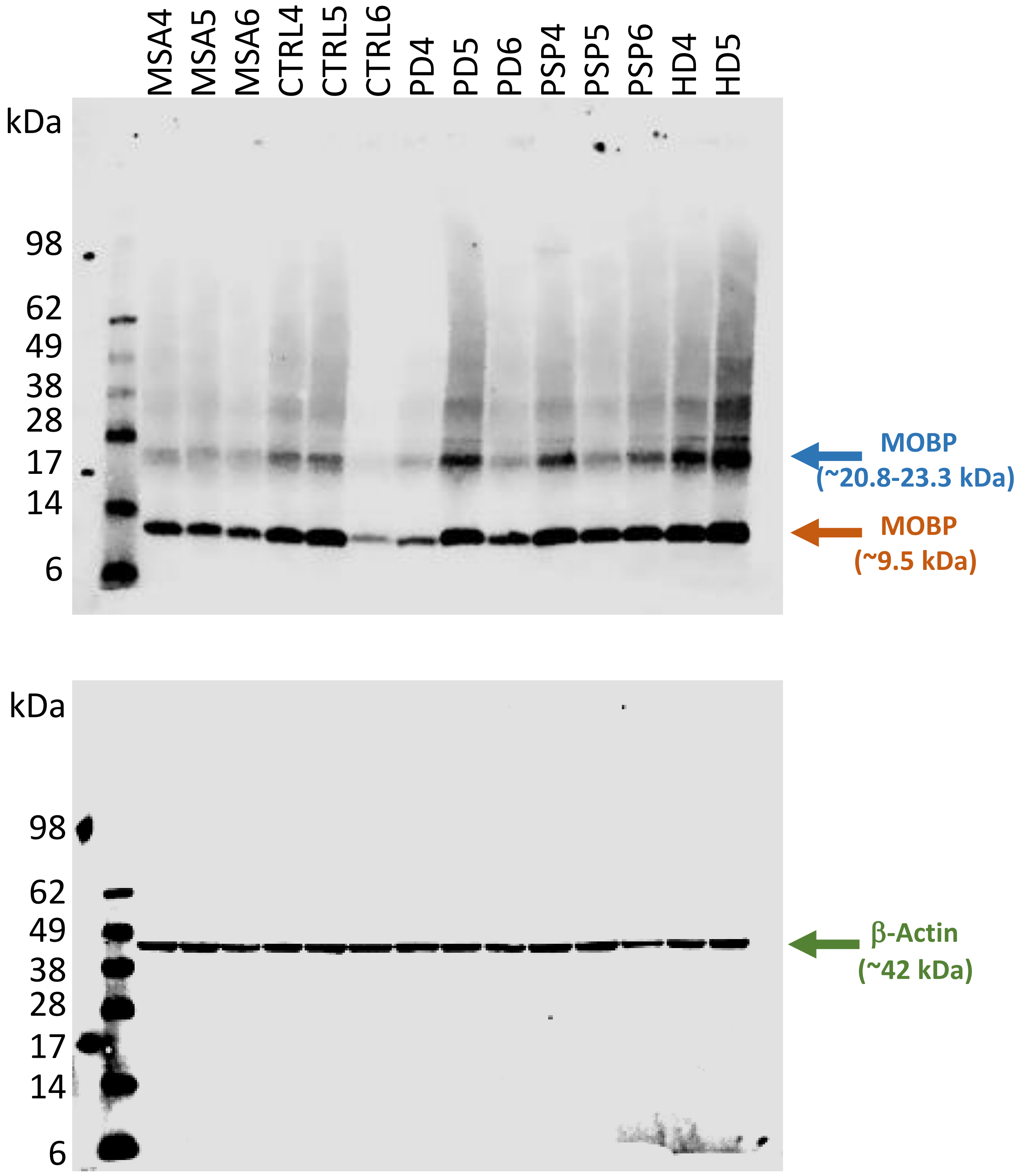
MSA4
MSA5
MSA6
CTRL4
CTRL5
CTRL6
PD4
PD5
PD6
PSP4
PSP5
PSP6
HD4
HD5
kDa
98
62
49
38
28
17
14
6
MOBP
(~20.8-23.3 kDa)
MOBP
(~9.5 kDa)
kDa
98
62
49
38
28
17
14
6
β-Actin
(~42 kDa)

d

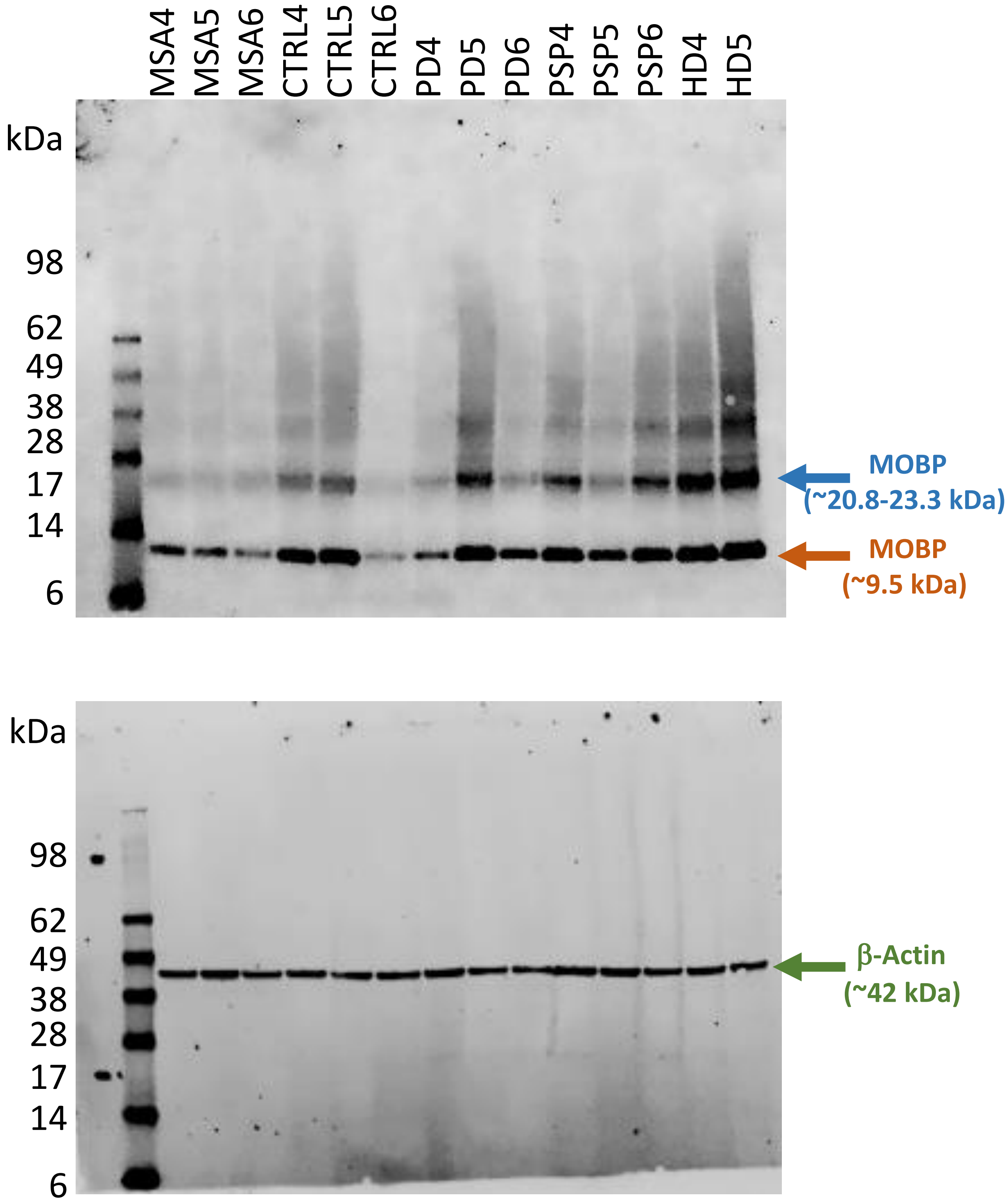

MSA4
MSA5
MSA6
CTRL4
CTRL5
CTRL6
PD4
PD5
PD6
PSP4
PSP5
PSP6
HD4
HD5
kDa
98
62
49
38
28
17
14
6
MOBP
(~20.8-23.3 kDa)
MOBP
(~9.5 kDa)
kDa
98
62
49
38
28
17
14
6
β-Actin
(~42 kDa)

Supplementary Figure 2: Original full length HIP1 immunoblots related to Figure 5. Immunoblots were labelled with anti-HIP1 (1:2,000; Abcam ab181238), anti-β-actin (1:5,000; Sigma A1978) and IRDye secondary antibodies (LiCor), and scanned on a LiCor Odyssey Fc. Samples were run in a total of 4 gels (**a-d**): **b** is a duplicate of **a**; **d** is a duplicate of **c**.

a

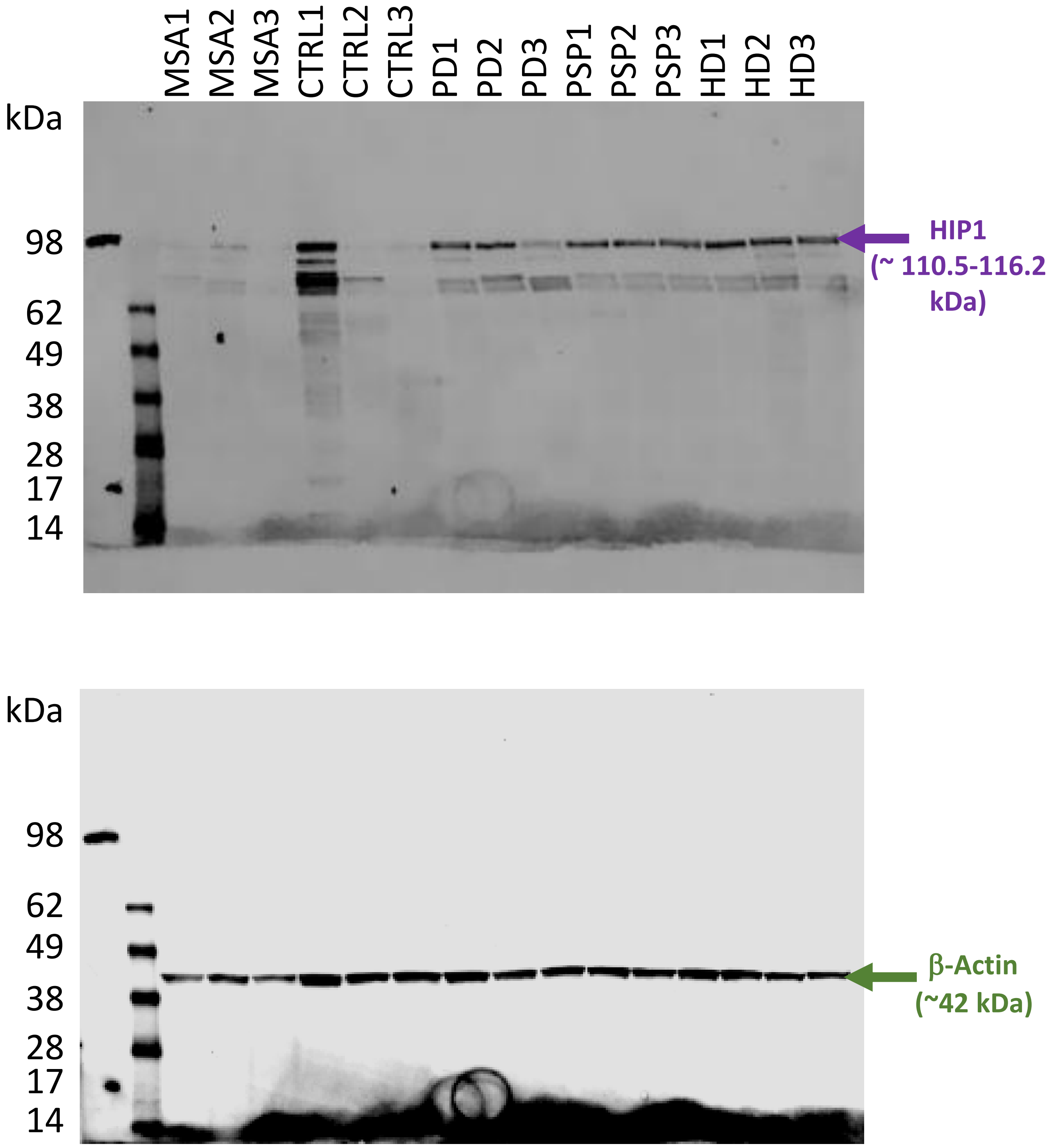

b

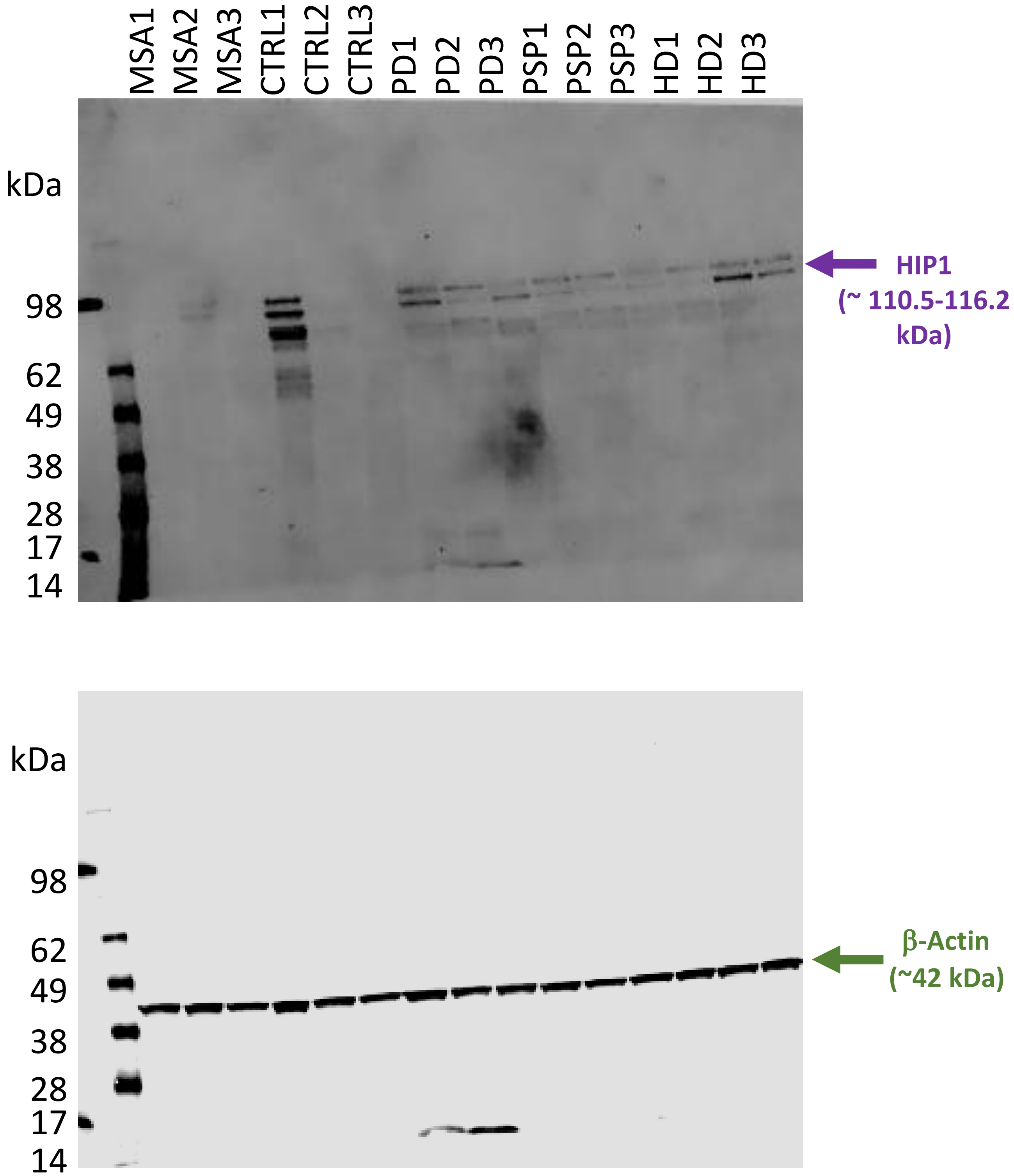
MSA1
MSA2
MSA3
CTRL1
CTRL2
CTRL3
PD1
PD2
PD3
PSP1
PSP2
PSP3
HD1
HD2
HD3
kDa
98
62
49
38
28
17
14
HIP1
(~ 110.5-116.2 kDa)
kDa
98
62
49
38
28
17
14
β-Actin
(~42 kDa)

c

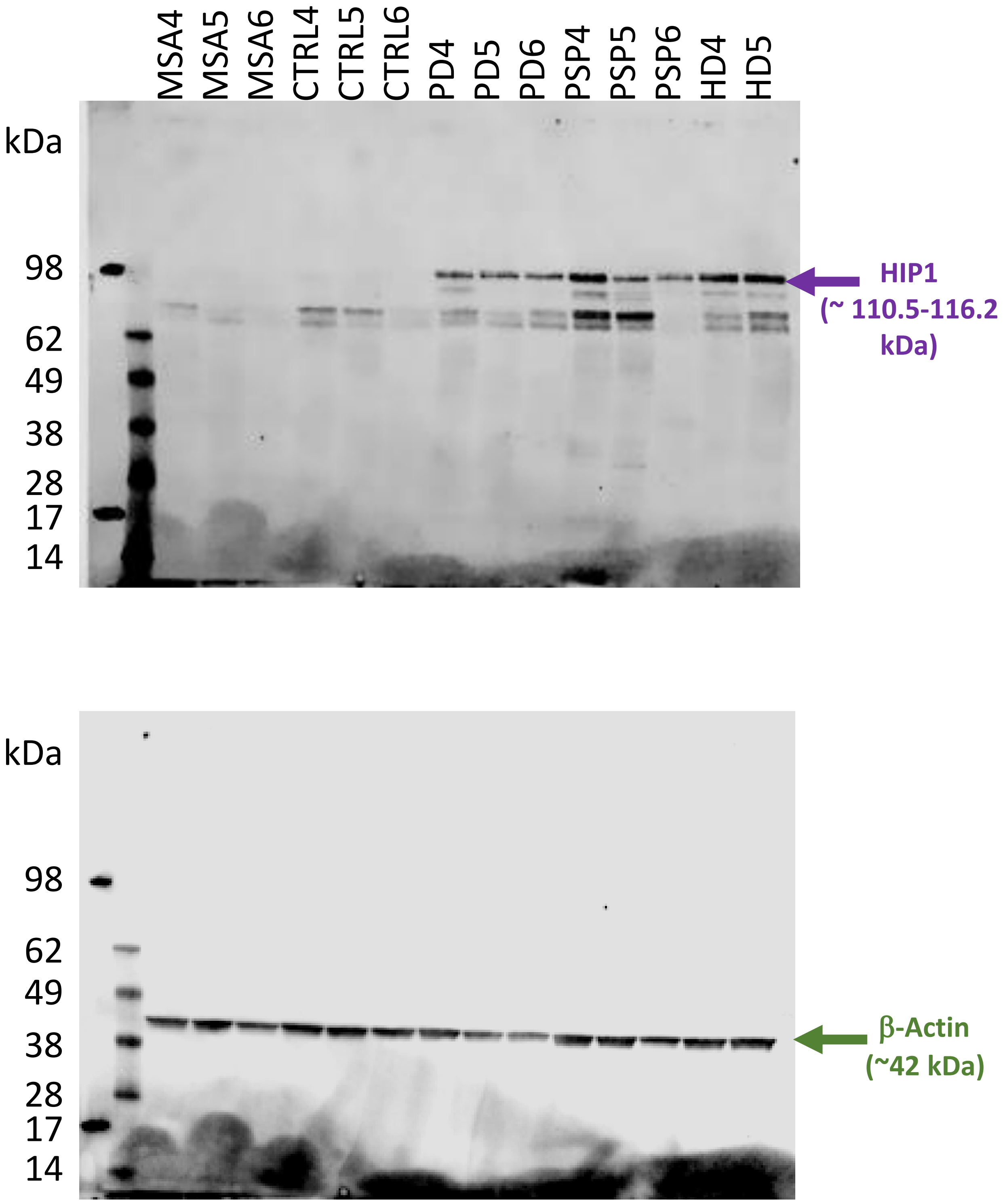
MSA4
MSA5
MSA6
CTRL4
CTRL5
CTRL6
PD4
PD5
PD6
PSP4
PSP5
PSP6
HD4
HD5
kDa
98
62
49
38
28
17
14
HIP1
(~ 110.5-116.2 kDa)
kDa
98
62
49
38
28
17
14
β-Actin
(~42 kDa)

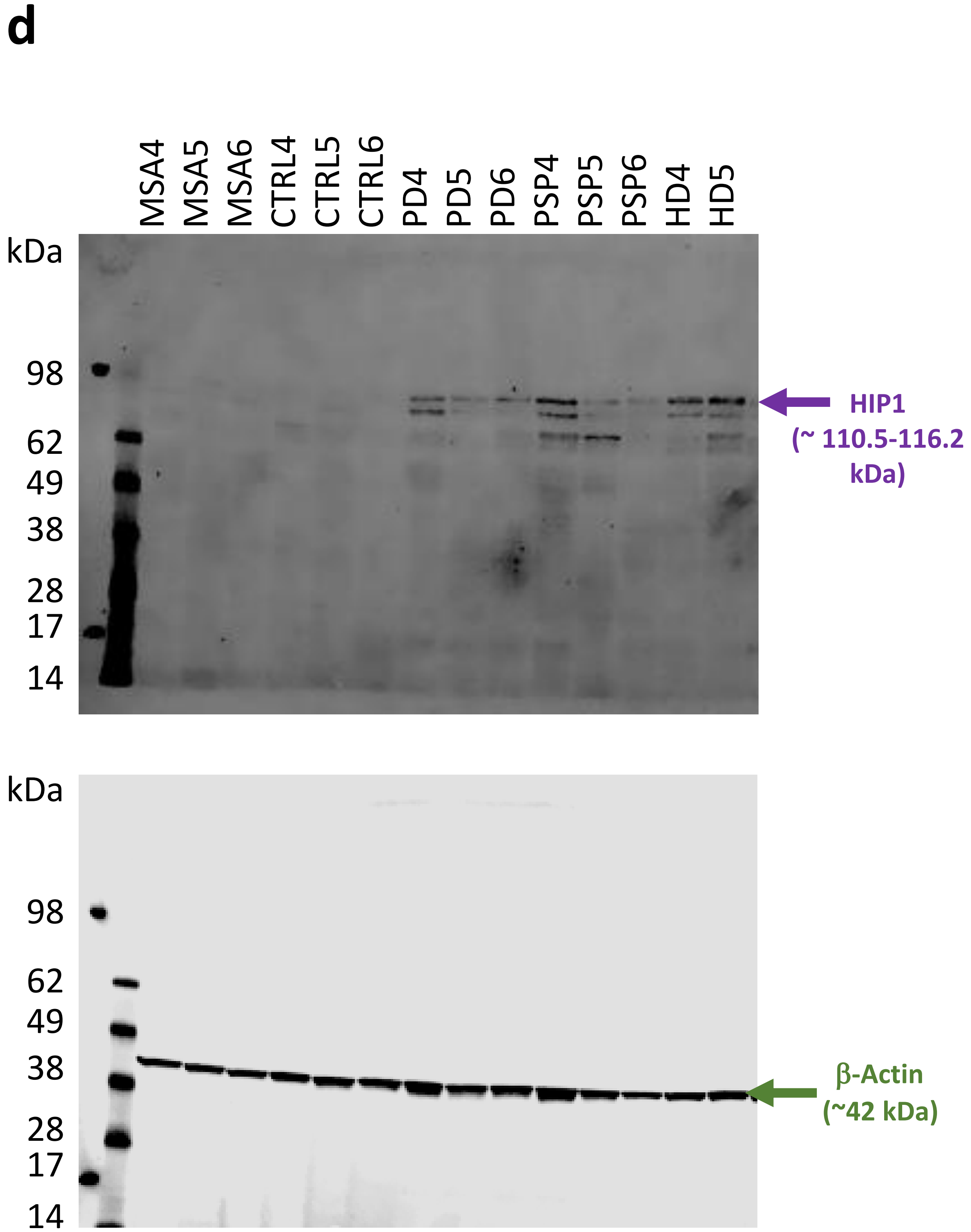
d
MSA4
MSA5
MSA6
CTRL4
CTRL5
CTRL6
PD4
PD5
PD6
PSP4
PSP5
PSP6
HD4
HD5
kDa
98
62
49
38
28
17
14
HIP1
(~ 110.5-116.2 kDa)
kDa
98
62
49
38
28
17
14
β-Actin
(~42 kDa)
