# Supplementary material for: MOBP and HIP1 in multiple system atrophy: New α‐synuclein partners in glial cytoplasmic inclusions implicated in the disease pathogenesis
Source: Neuropathol Appl Neurobiol. 2021 Jan 19;47(5):640–52. doi: 10.1111/nan.12688 (PMC8219819; doi:10.1111/nan.12688)
Supplement: Supplementary file 2 — Fig S3‐S7 [file NAN-47-640-s005.pdf]

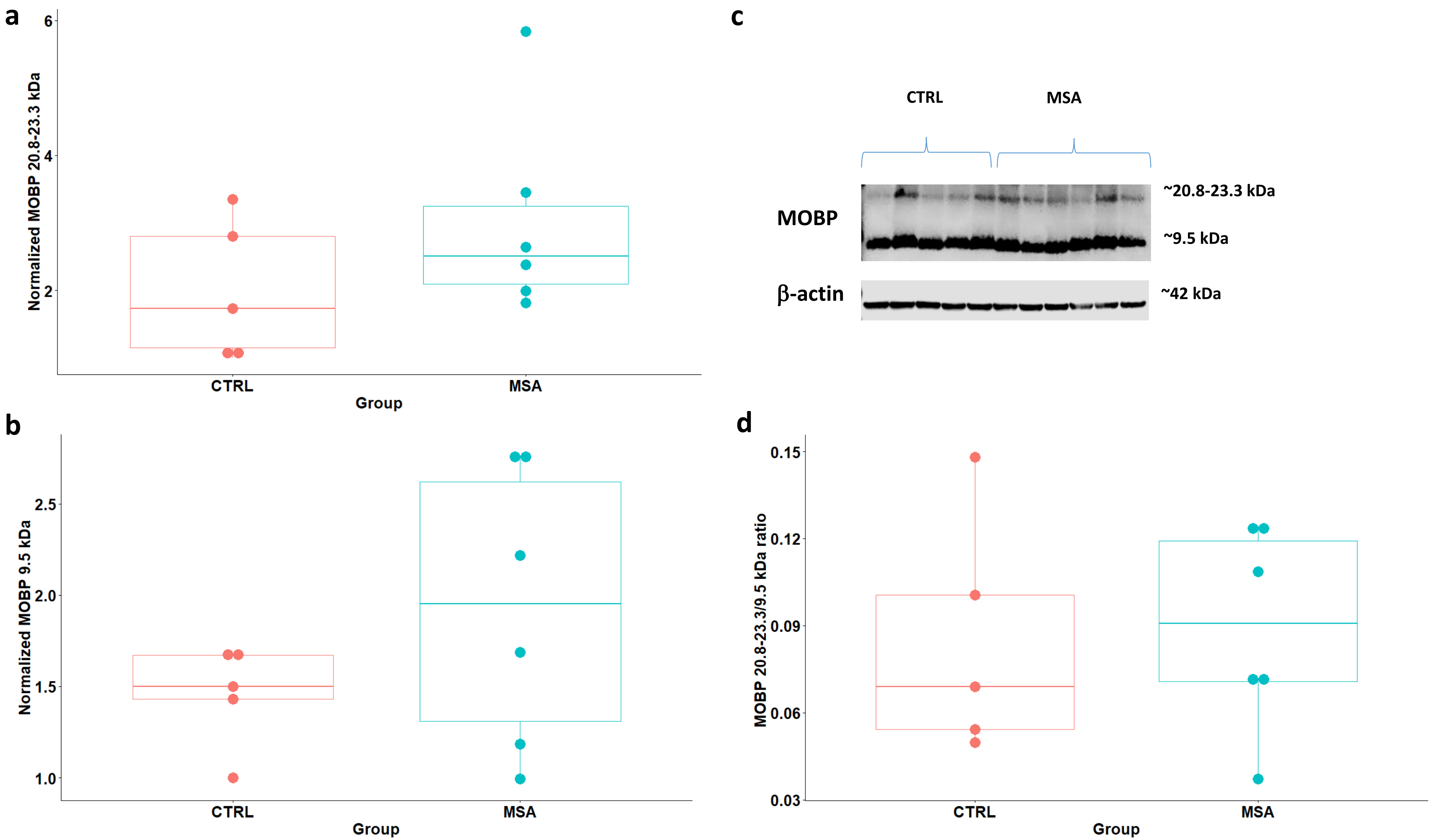

Supplementary Fig. 3: Western blot detection of MOBP in occipital lobe white matter lysates (N = 11). Immunoblots were labelled with anti-MOBP (Atlas Antibodies HPA035152, 1:250), anti-β-actin (Sigma A1978, 1:5,000) and IRDye secondary antibodies (LiCor), and scanned on a LiCor Odyssey Fc (c). Intensities for the 20.8-23.3 kDa (isoforms b and a) and 9.5 kDa (isoform c) bands, respectively, were derived from raw scan data, analysed on Image Studio (LiCor), and normalised against β -actin band for each sample as housekeeping gene (a-b). Analysis of the ratio between levels of 20.8-23.3 kDa (isoforms b and a) over 9.5 kDa (isoform c) bands (d). CTRL: healthy controls; MSA: multiple system atrophy.

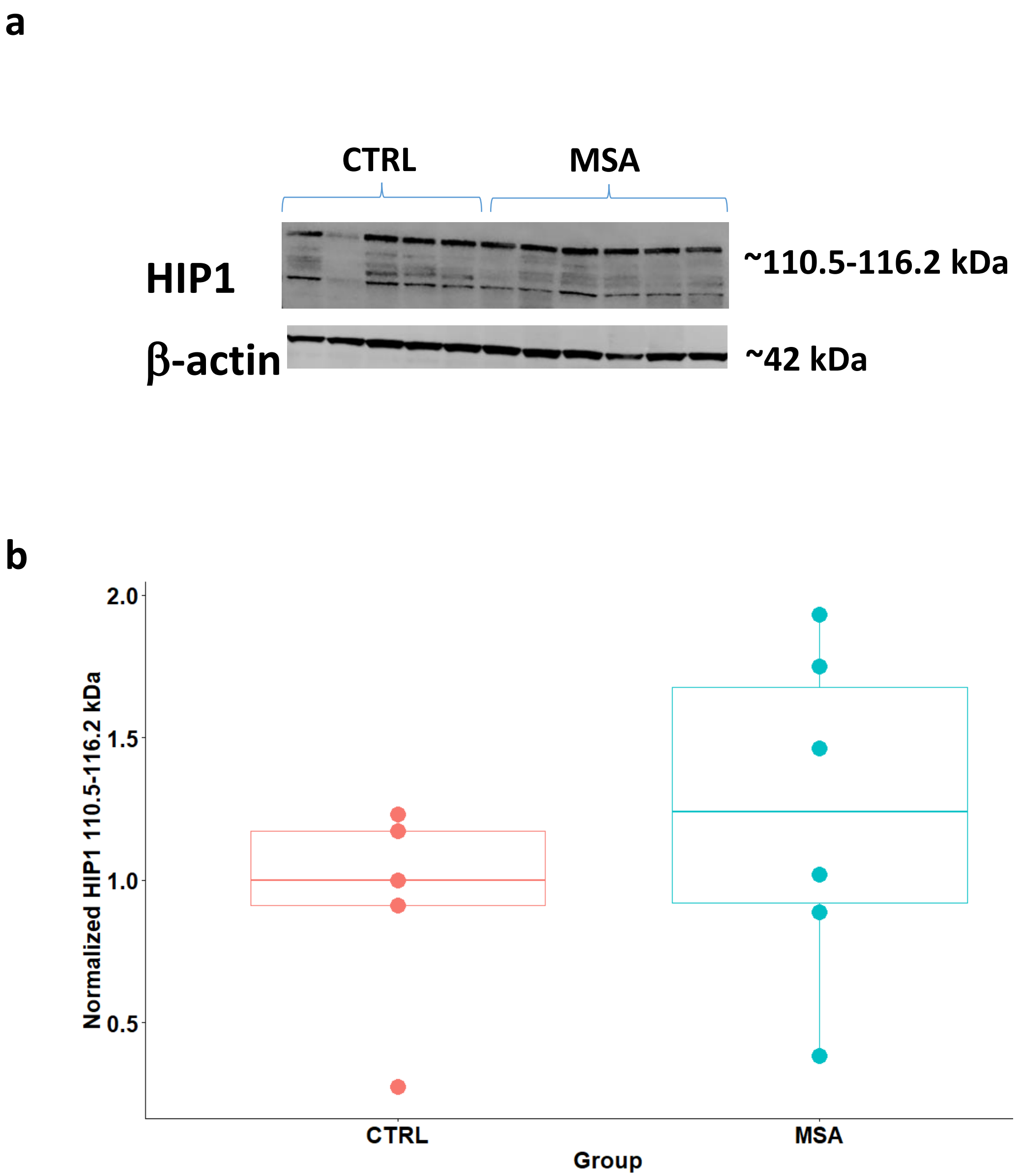

Supplementary Fig. 4: Western blot detection of HIP1 in occipital lobe white matter lysates (N = 11). Immunoblots were labelled with anti-HIP1 (Abcam ab181238, 1:2,000), anti-β-actin (Sigma A1978, 1:5,000) and IRDye secondary antibodies (LiCor), and scanned on a LiCor Odyssey Fc (a). Band intensities were derived from raw scan data and analysed on Image Studio (LiCor). HIP1 band intensities normalised against β-actin as housekeeping gene (b). CTRL: healthy controls; MSA: multiple system atrophy.

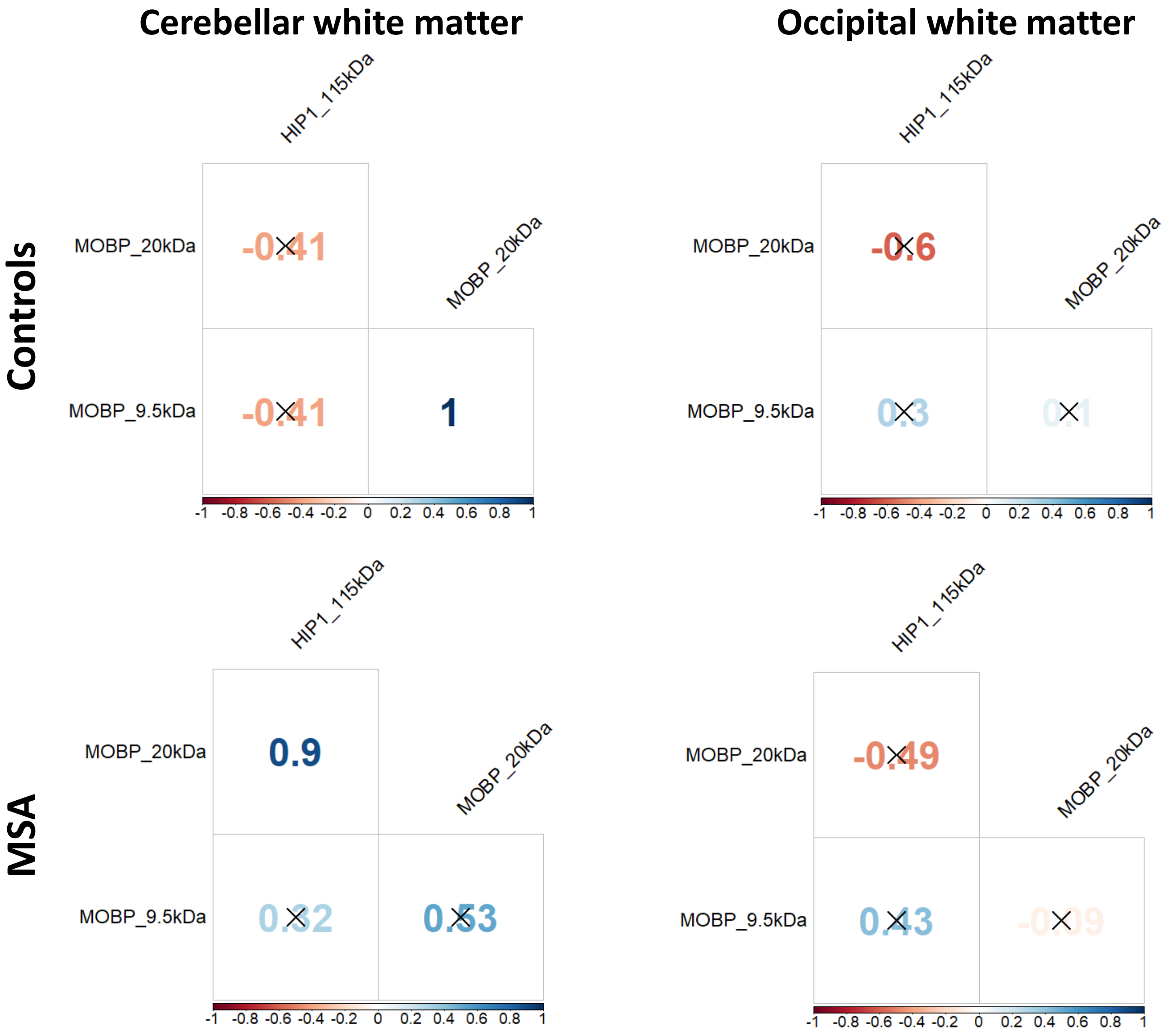

Supplementary Fig. 5: Graphical representation of the correlation matrix between MOBP and HIP1 protein levels. Positive correlation coefficients are displayed in blue and negative correlation coefficients in red colour. Non-significant p-values ($p > 0.05$) are represented by a cross. MOBP_20kDa represents the 20.8-23.3 kDa MOBP isoforms.

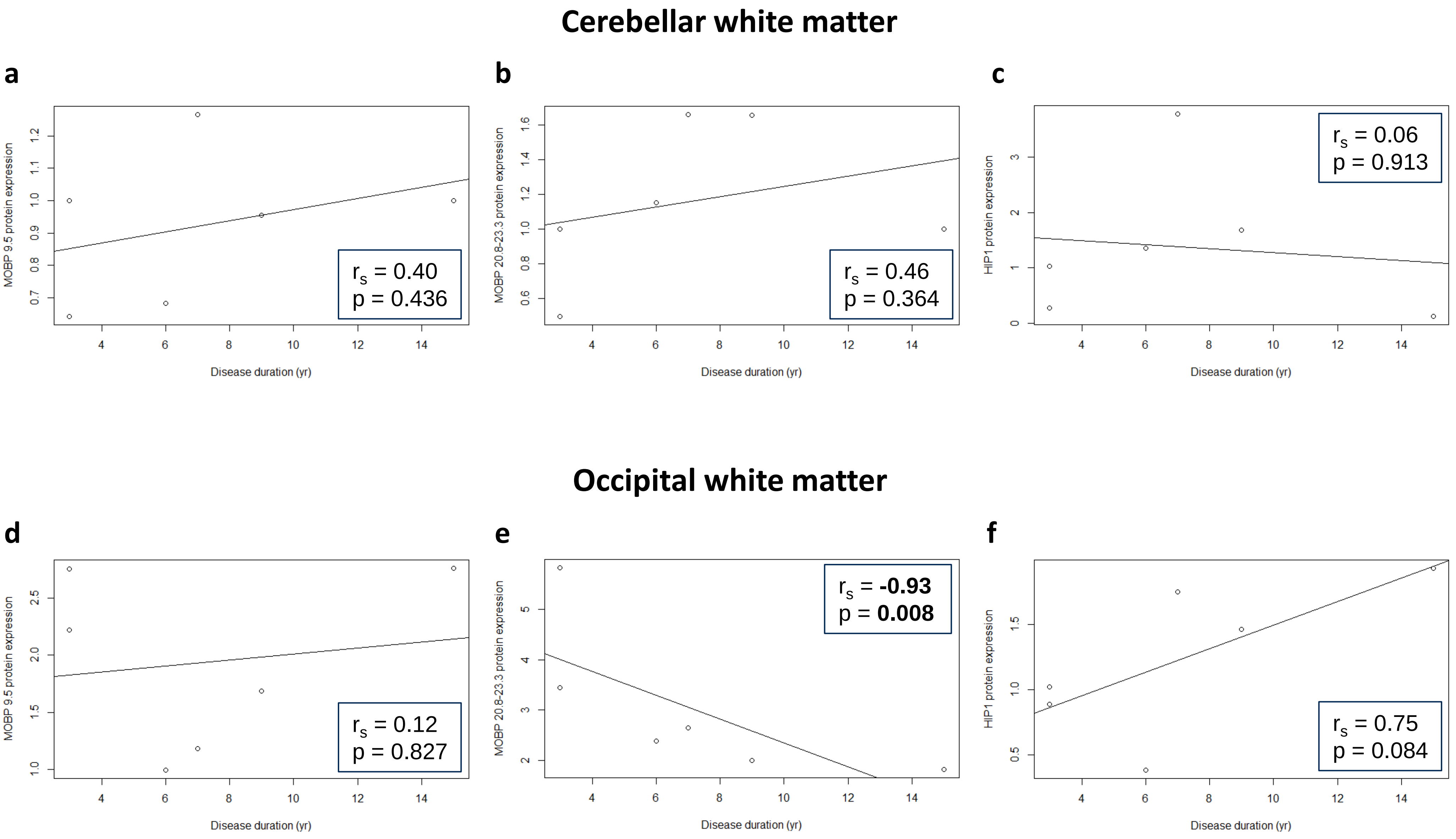

Supplementary Fig. 6: Scatterplots illustrating the relationship between MOBP and HIP1 protein levels and the MSA disease duration. (a, d) MOBP 9.5 kDa isoform; (b, e) MOBP 20.8-23.3 kDa isoforms; (c, f) HIP1. r_s (Spearman correlation coefficients) with significant p-values ($p < 0.05$) are highlighted in bold.

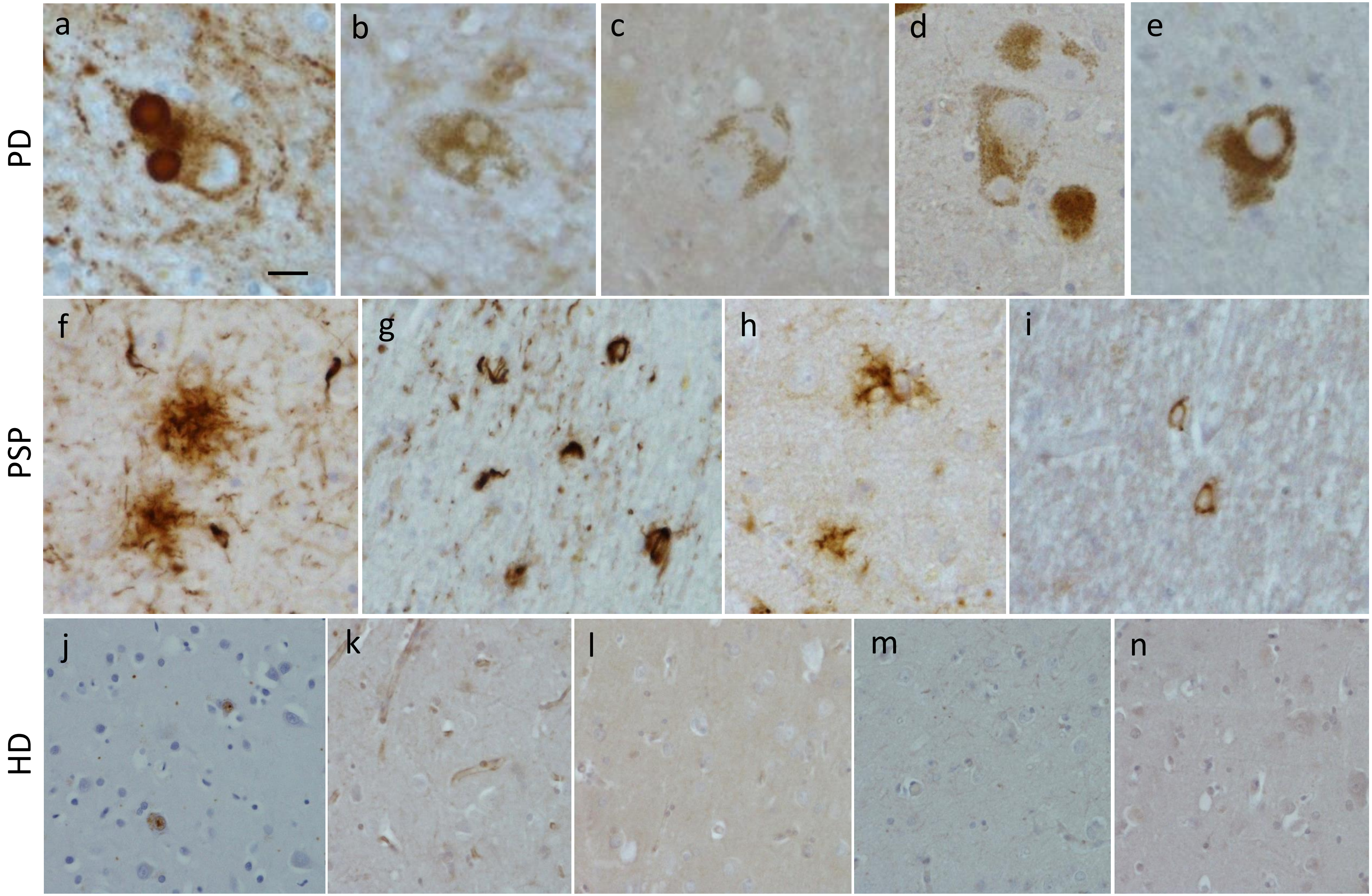

Supplementary Fig. 7: Immunoreactivity of MOBP and HIP in PD (N = 3), PSP (N = 3) and HD patients (N = 3). Immunohistochemical analysis was carried out with two HIP1 antibodies (NB300-203 and ab181238) and two MOBP antibodies (HPA035152 and bs-11184R) in PD midbrain and frontal cortex for PSP and HD. In PD, Lewy bodies are shown with alpha-synuclein immunohistochemistry (a), but were negative for both HIP1 (b and c) and MOBP (d and e). In PSP, AT8 immunohistochemistry highlights tufted astrocytes in the grey matter (f) and coiled bodies in oligodendrocytes in the white matter (g). These pathological inclusions were also present with one of the HIP1 antibodies (ab181238), staining tufted astrocytes in the grey matter (h) and coiled bodies in the white matter (i). All the remaining antibodies were negative in PSP. In HD, all antibodies used were negative for the intranuclear inclusions seen using the 1C2 antibody (j). Bar in a represents 10µm in a-e; 15µm in f-I and 30µm in j-m.
